# Human neutrophil extracellular traps do not impair *in vitro Toxoplasma gondii* infection

**DOI:** 10.3389/fimmu.2023.1282278

**Published:** 2023-12-05

**Authors:** Isabela S. Macedo, Flávio A. Lara, Helene S. Barbosa, Elvira M. Saraiva, Rubem F. S. Menna-Barreto, Rafael M. Mariante

**Affiliations:** ^1^ Laboratório de Biologia Estrutural, Instituto Oswaldo Cruz, Fiocruz, Rio de Janeiro, Brazil; ^2^ Laboratório de Microbiologia Celular, Instituto Oswaldo Cruz, Fiocruz, Rio de Janeiro, Brazil; ^3^ Laboratório de Imunobiologia das Leishmanioses, Instituto de Microbiologia Paulo de Góes, Universidade Federal do Rio de Janeiro, Rio de Janeiro, Brazil; ^4^ Laboratório de Biologia Celular, Instituto Oswaldo Cruz, Fiocruz, Rio de Janeiro, Brazil

**Keywords:** NET, *Toxoplasma gondii*, human neutrophils, classic/rapid NETs, infectivity, viability, entrapment, live cell imaging

## Abstract

**Introduction:**

*Toxoplasma gondii*, responsible for causing toxoplasmosis, is a prevalent food and waterborne pathogen worldwide. It commonly infects warm-blooded animals and affects more than a third of the global human population. Once ingested, the parasite enters the host’s small intestine and rapidly disseminates throughout the body via the bloodstream, infiltrating various tissues. Leukocyte-driven responses are vital against *T. gondii*, with neutrophils playing a dual role: swiftly recruited to infection sites, releasing inflammatory mediators, and serving as a replication hub and Trojan horses, aiding parasite spread. Neutrophils from various hosts release extracellular traps (NETs) against the protozoan. However, gaps persist regarding the mechanisms of NETs production to parasite and their significance in infection control. This study investigates the interplay between human neutrophils and *T. gondii*, exploring dynamics, key molecules, and signaling pathways involved in NETs production upon protozoan challenge.

**Methods and Results:**

Using confocal and electron microscopy, live cell imaging, pharmacological inhibitors, and DNA quantification assays, we find that human neutrophils promptly release both classical and rapid NETs upon pathogen stimulation. The NETs structure exhibits diverse phenotypes over time and is consistently associated with microorganisms. Mechanisms involve neutrophil elastase and peptidylarginine deiminase, along with intracellular calcium signaling and the PI3K pathway. Unexpectedly, human traps do not diminish viability or infectivity, but potentially aid in capturing parasites for subsequent neutrophil phagocytosis and elimination.

**Discussion:**

By revealing NETs formation mechanisms and their nuanced impact on *T. gondii* infection dynamics, our findings contribute to broader insights into host-pathogen relationships.

## 1 Introduction

*Toxoplasma gondii*, the causative agent of toxoplasmosis, stands out as an important global food and waterborne pathogen. Its ability to infect a wide range of warm-blooded animals, including humans, underscores its global prevalence, with an estimated chronic infection affecting more than 2 billion people (1). Transmission primarily occurs through the ingestion of contaminated water, vegetables, or the consumption of undercooked meat that harbors the parasite (2). Although often resulting in asymptomatic infections, it poses a significant risk to immunocompromised individuals, who are susceptible to developing retinochoroiditis, myocarditis, pneumonitis, and especially encephalitis (3). Pregnant women also face greater vulnerability, as the parasites deftly cross the placental barrier, culminating in severe damage to the developing fetus (4). Pets and livestock are also affected by congenital toxoplasmosis. In livestock, it leads to significant economic losses due to reproductive problems such as miscarriages, fetal malformations, premature births and stillbirths (5).

After oral ingestion, *T. gondii* invades the host’s small intestine and spreads rapidly through the bloodstream to infiltrate a variety of tissues, including muscles, brain, eyes, liver, placenta, and lungs (6). Protective cellular immune responses orchestrated by leukocytes are essential to combat the protozoan and critical to establishing chronic toxoplasmosis (7, 8). Neutrophils, as prominent leukocytes, play a dual role in the infection. On the positive side, neutrophils are rapidly recruited to sites of infection, releasing inflammatory mediators that bolster the immune response (9–12). Conversely, on the negative side, they are recognized as susceptible to and function as a replication hub for *T. gondii*, also operating as Trojan horses that aid in the spread of the pathogen through the intestinal tract (13–15).

Among the various mechanisms neutrophils employ to combat pathogenic microorganisms, the generation of neutrophil extracellular traps (NETs) has attracted significant attention over the last two decades due to their ability to immobilize and kill microbes of different classes. The production of NETs involves the release of chromatin adorned with antimicrobial peptides and proteases. This process can occur either in a classical manner, after several minutes to hours of exposure to the stimulus, leading to plasma membrane rupture and neutrophil death, or in a rapid manner, after a few minutes of neutrophil stimulation, resulting in the preservation of viable and functional cytoplasts (16–20). The mechanisms that govern the production of NETs are multifactorial and vary according to the host of origin of the neutrophils, the stimulating agent and the time of exposure to the stimulus (21, 22 23, 24).

Currently, we know that neutrophils from various hosts release NETs in response to *T. gondii*. Abi Abdallah et al. (25) first showed that human and murine neutrophils release classical NETs against the parasite, and that murine traps impact parasite viability and infectivity. In the subsequent years, we and others have provided evidence of NETs generation to *T. gondii* released by neutrophils from different hosts, highlighting diverse signaling pathways (26–36). Recently, human neutrophil traps triggered by the protozoan regained attention after a decade from Abi Abdallah’s work, with Miranda et al. (37) uncovering that NET release depends on active parasite invasion and involves mitochondrial metabolism, gasdermin D, neutrophil elastase (NE), and reactive oxygen species (ROS). Moreover, the authors demonstrated *in vitro* the importance of NETs in influencing the activation and migration of neutrophils, as well as in amplifying the adaptive immune response by recruiting lymphocytes and producing cytokines crucial to infection (37).

Here, we delve deeper into the intricate interplay between human neutrophils and *T. gondii*. We explore the dynamics of NET formation against the pathogen, scrutinizing the involvement of key molecules associated with chromatin decondensation, such as peptidylarginine deiminases (PAD) and myeloperoxidase (MPO), along with NE, in rapid and classical NETs. Furthermore, we extend our investigation to intracellular calcium, phosphoinositide 3-kinases (PI3K), and MAPK/ERK signaling pathways, seeking a more comprehensive understanding of the signaling cascades underlying NET production upon parasite challenge. Ultimately, we aim to decipher whether human traps manifest as an effective mechanism against the protozoan, potentially influencing its viability and/or its ability to infect the host cell – a facet mirroring observations seen with NETs generated by neutrophils from alternate hosts (25–28, 32).

## 2 Materials and methods

### 2.1 Reagents

Propidium iodide and DABCO were obtained from Sigma-Aldrich (USA). Quant-iT PicoGreen dsDNA Assay Kit, Amplex Red Hydrogen Peroxide/Peroxidase Assay Kit, and PrestoBlue Cell Viability Reagent were acquired from Invitrogen (Thermo Fisher Scientific, USA).

We used the following pharmacological inhibitors/inducers in the study: phorbol 12-myristate 13-acetate (PMA; 100 nM), cytochalasin D (CytD; 10 µg/mL), PD98059 (PD98; MEK inhibitor; 60 µM), and 3-Methyladenine (3-MA; class III PI3K Vps34 and autophagy inhibitor; 5 mM) from Sigma-Aldrich; neutrophil elastase inhibitor III (NEi; MeOSuc-AAPV-CMK; 10 µg/mL), myeloperoxidase inhibitor I (MPOi; 600 nM), and BAPTA-AM (BAPTA; [Ca^2+^]i chelator; 10 µM) from Calbiochem (USA); IC87114 (IC87; selective inhibitor of PI3Kδ; 1 µM) and chloroamidine (Cl-A; PAD inhibitor; 12 μM) from Cayman Chemical (USA); and AS605240 (AS60; selective inhibitor of PI3Kγ; 10 µM) from Tocris Bioscience (UK).

The following antibodies were used in this study: mouse anti-histone H1 monoclonal (sc-8030; 1:100) from Santa Cruz Biotechnology (USA); rabbit anti-myeloperoxidase polyclonal (PA5-16672; 1:200), mouse anti-toxoplasma monoclonal (MA1-83499; 1:100), Goat anti-Mouse Alexa Fluor 488 (A11001; 1:2000), Goat anti-Mouse Alexa Fluor 546 (A11003; 1:400), and Goat anti-Rabbit Alexa Fluor 594 (A11037; 1:800) from Invitrogen.

### 2.2 Neutrophils and parasites cultures

Neutrophils were isolated by density gradient centrifugation as previously described (32). Afterwards, cells were washed with PBS, suspended in RPMI 1640 medium supplemented with 1% fetal calf serum, 200 mM glutamine and 1% penicillin-streptomycin antibiotics solution, and kept on ice until use. The degree of purity of the neutrophils, assessed by staining the cytocentrifugation slides with May-Grunwald-Giemsa, was greater than 88%.

Tachyzoites of *T. gondii* from RH strain are routinely maintained in Swiss Webster mice, being isolated and cultured essentially as previously described (32). Parasites fixed in paraformaldehyde have also been used to stimulate neutrophils. For this purpose, the tachyzoites were fixed with 4% paraformaldehyde for 60 min at room temperature and then washed three times with PBS. The parasites were resuspended in PBS containing a 1% penicillin-streptomycin antibiotics solution, counted in a hemocytometer, and kept at 4°C until use.

### 2.3 NET induction/inhibition assays

To induce NET production, neutrophils (1.5 x 10^5^ cells per well) were seeded in 96-well plates and incubated with 100 nM PMA, live *T. gondii* tachyzoites or paraformaldehyde-fixed tachyzoites (5:1 parasite/neutrophil ratio) for 15 or 180 min at 37°C, 5% CO_2_. For inhibition assays, neutrophils were treated for 20 min with the pharmacological inhibitors listed above before adding *T. gondii* tachyzoites. Then, the supernatants were collected, centrifuged (1,500 × *g* for 10 min) and stored at 80°C until use.

For the parasite infectivity assay (see below), NETs were generated in 6-well plates. Neutrophils (2 x 10^6^ cells per well) were stimulated (conditioned medium with NET, CM NET) or not (conditioned medium control, CM CTR) with tachyzoites as described above. For some experiments, NET-enriched supernatants were filtered with 0.22 µm pore size membrane to retain NET fibers (CM NET FL). NET-derived DNA from the supernatants was quantified with the PicoGreen assay as described below.

### 2.4 Cytotoxicity of inhibitors on neutrophils

Cytotoxicity of the drugs towards neutrophils was evaluated with PrestoBlue Cell Viability Reagent according to the manufacturer’s instructions. Briefly, neutrophils were incubated with inhibitors as described above and held at 37°C, 5% CO_2_, for 180 min. PrestoBlue was added 20 min before the end of the incubation time and analysis was performed in a SpectraMax M2 microplate reader (Molecular Devices, USA) using excitation/emission wavelengths of 560/590 nm. Data are represented as percentage of untreated control.

### 2.5 Quantification of NET-derived DNA

Cell culture supernatants were distributed into opaque 96-well plates and NET-derived DNA was quantified using the Quant-iT PicoGreen dsDNA Assay Kit according to the manufacturer’s instructions. Analysis was performed in a SpectraMax M3 microplate reader (Molecular Devices) using excitation/emission wavelengths of 480/520 nm. The standard concentration curve was made with herring sperm DNA.

### 2.6 Immunofluorescence assay

Neutrophils (2.5 x 10^5^ cells per well) were seeded in 24-well plates with coverslips and incubated or not with *T. gondii* tachyzoites (5:1 parasites/neutrophil ratio) for 15 or 180 min at 37°C, 5% CO_2_. Then, the cells were fixed with 4% paraformaldehyde for 30 min at room temperature and carefully washed with PBS. Coverslips were incubated overnight at 4°C with anti-MPO, anti-Histone H1 or anti-*T. gondii*, followed by secondary antibodies. Finally, coverslips were stained with DAPI (1µg/mL) and mounted on slides with DABCO antifading agent. Image acquisition was performed using a Leica TCS SP8 confocal microscope (Leica, Germany). Analysis and processing were done in Leica LAS X Office 1.4 (Leica, Germany) and Adobe Photoshop 24.7 (Abode, USA) software.

### 2.7 Scanning electron microscopy

Neutrophils (2.5 x 10^5^ cells per well) were seeded in 24-well plates with coverslips and stimulated with *T. gondii* tachyzoites (5:1 parasites/neutrophil ratio) for 15 or 180 min at 37°C, 5% CO_2_. Cells were fixed for 1 h at room temperature with 2.5% glutaraldehyde in 0.1 M sodium cacodylate buffer (pH 7.2) containing 3.5% sucrose and 2.5 mM CaCl_2_. After washing, the cells were post-fixed with 1% osmium tetroxide and 0.8% potassium ferricyanide for 1 h at room temperature and dehydrated in ethanol at increasing concentrations. Then, the specimens were critical-point dried, covered with gold, and observed in a JEOL-JSM-6390LV scanning electron microscope (JEOL, Japan) of the Rudolf Barth Electron Microscopy Platform (Instituto Oswaldo Cruz, Fiocruz, Brazil).

### 2.8 Transmission electron microscopy

Neutrophils (1.25 x 10^6^ cells per well) were seeded in 6-well plates and stimulated with *T. gondii* tachyzoites (5:1 parasites/neutrophil ratio) for 15, 180 or 240 min at 37°C, 5% CO_2_. Cells were fixed and post-fixed as described above and dehydrated in acetone at increasing concentrations. Samples were embedded in PolyBed 812 resin (Polysciences, UK) and polymerized for 72 h at 60°C. Ultrathin sections were obtained in an Ultracut Ultramicrotome (Leica, Austria) and collected on copper grids. The sections were contrasted in 1% uranyl acetate and lead citrate and the grids examined using Hitachi HT 7800 (Hitachi High-Tech, Japan) or Jeol 1011 (Jeol, Japan) transmission electron microscopes at the Centro Nacional de Biologia Estrutural e Bioimagem (CENABIO, UFRJ, Brazil) or at the Rudolf Barth Electron Microscopy Platform (Instituto Oswaldo Cruz, Fiocruz, Brazil).

### 2.9 Measurement of hydrogen peroxide release

The release of hydrogen peroxide (H_2_O_2_) by neutrophils was determined using Amplex Red Hydrogen Peroxide/Peroxidase Assay Kit according to the manufacturer’s instructions. Briefly, neutrophils (2 x 10^4^ cells per well) diluted in Krebs-Ringer phosphate (KRPG, 145 mM NaCl, 5.7 mM sodium phosphate, 4.86 mM KCl, 0.54 mM CaCl_2_, 1.22 mM MgSO_4_, 5.5 mM glucose, pH 7.4) were incubated with *T. gondii* tachyzoites (5:1 parasites/neutrophil ratio) in the presence of 50 μM Amplex Red reagent and 0.1 U/mL horseradish peroxidase in KRPG. The rate of formation of fluorescent resofurin, the reaction oxidation product, was recorded at 37°C for 180 min in a SpectraMax M3 microplate reader (Molecular Devices) using excitation/emission wavelengths of 530/590 nm. Calibration was performed using hydrogen peroxide as standard.

### 2.10 Parasite infectivity assay

*T. gondii* tachyzoites were incubated in supernatants (CM CTR, CM NET or CM NET FLTR) for 180 min at 37°C, 5% CO_2_. Then parasites were used to infect Vero cell monolayers (1:1 parasite/Vero ratio) on coverslips for 180 min. Cells were then washed to remove non-internalized parasites and kept at 37°C, 5% CO_2_, for 21 h. After 24 h of interaction, the coverslips were stained with Giemsa and observed in a Zeiss Axio Imager.A2 microscope. At least 200 cells were randomly counted on each slide, and the percentage of infected cells and the number of parasites per cell were calculated.

### 2.11 Live cell imaging

NET production over time and its effect on parasite entrapment/death were investigated by live cell imaging as previously described (32). Briefly, neutrophils (5 x 10^5^) were seeded onto 35 mm CELLview plates (Greiner Bio-One, Brazil) and allowed to adhere for 30 min at 37°C. Parasites were added (5:1 parasite/neutrophil ratio) in the presence of propidium iodide. NET release and parasite entrapment/death were monitored for 4.5 h on a Zeiss Axio Observer Z1 microscope equipped with Definitive Focus and a HMR Axiocam monochrome camera, with temperature stabilized at 37°C. Spontaneous death of parasites in NET-enriched supernatant or culture medium alone were also evaluated as control conditions. Image acquisition and analysis were performed using Zen 3.3 Blue Edition (Zeiss, Germany) and ImageJ 1.52 (National Institutes of Health, USA), respectively.

### 2.12 Statistical data analysis

Data are presented as individual samples or as mean ± SD values. The Kolmogorov-Smirnov test was used to assess the normality of the data. Comparisons between two groups were performed using the paired nonparametric Wilcoxon matched-pairs signed rank test. To compare more than two groups, we used One-way repeated measures ANOVA with Greenhouse-Geisser correction and Tukey’s multiple comparisons test or, for nonparametric data, the Friedman test with Dunn’s multiple comparisons test. Two-way ANOVA with Sidak’s multiple comparisons test was used for the analysis of H_2_O_2_ production. Differences with *p* < 0.05 were considered significant. We conducted all analyses using GraphPad Prism 8 software (GraphPad, USA).

## 3 Results

### 3.1 Human neutrophils release classical and rapid NETs triggered by *T. gondii*

**Figure 1:**
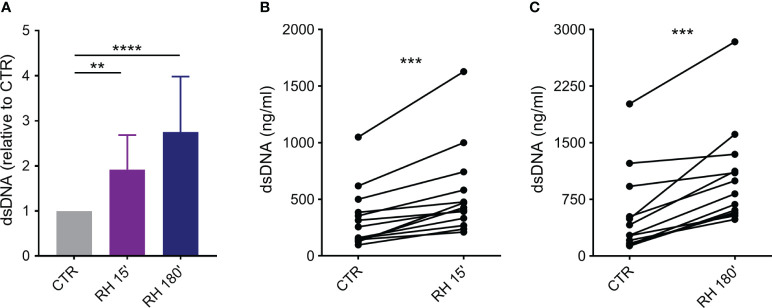
*T. gondii* induce rapid and classical NETs release from human neutrophils. Neutrophils from healthy donors were incubated for 15 or 180 min in the absence (CTR) or presence of tachyzoites from RH strain (5:1 parasites:neutrophil ratio). Supernatants were collected and released dsDNA was quantified with PicoGreen Kit. **(A)** Results are shown as means (SD) relative to CTR (*n* = 13). **(B, C)** Interdonor variations in dsDNA release after incubation with parasites (*n* = 13). ** *p* < 0.01; *** *p* < 0.001; **** *p* < 0.0001.

Initially, we investigated whether human neutrophils would be prone to release classical and rapid NETs in response to tachyzoites of the virulent RH strain of *T. gondii*. Positive controls of NET release were performed by incubating neutrophils with PMA, a known protein kinase C (PKC) activator and potent inducer of classical NET (23). As expected, PMA induced a significant increase in NET release when compared to untreated cells (**Supplementary Figure 1**). After interacting with parasites for 15 or 180 min (5:1 parasite:neutrophil ratio), neutrophils released rapid and classical NETs (**Figure 1A**). The amount of dsDNA released in the culture supernatants increased as a function of time (mean (SD) range of 1.92 ± 0.76 and 2.75 ± 1.23 times for 15 and 180 min, respectively, in relation to non-stimulated control). In addition, neutrophils from each donor responded by increasing NET release triggered by *T. gondii* in both time points (**Figures 1B, C**).

**Figure 2:**
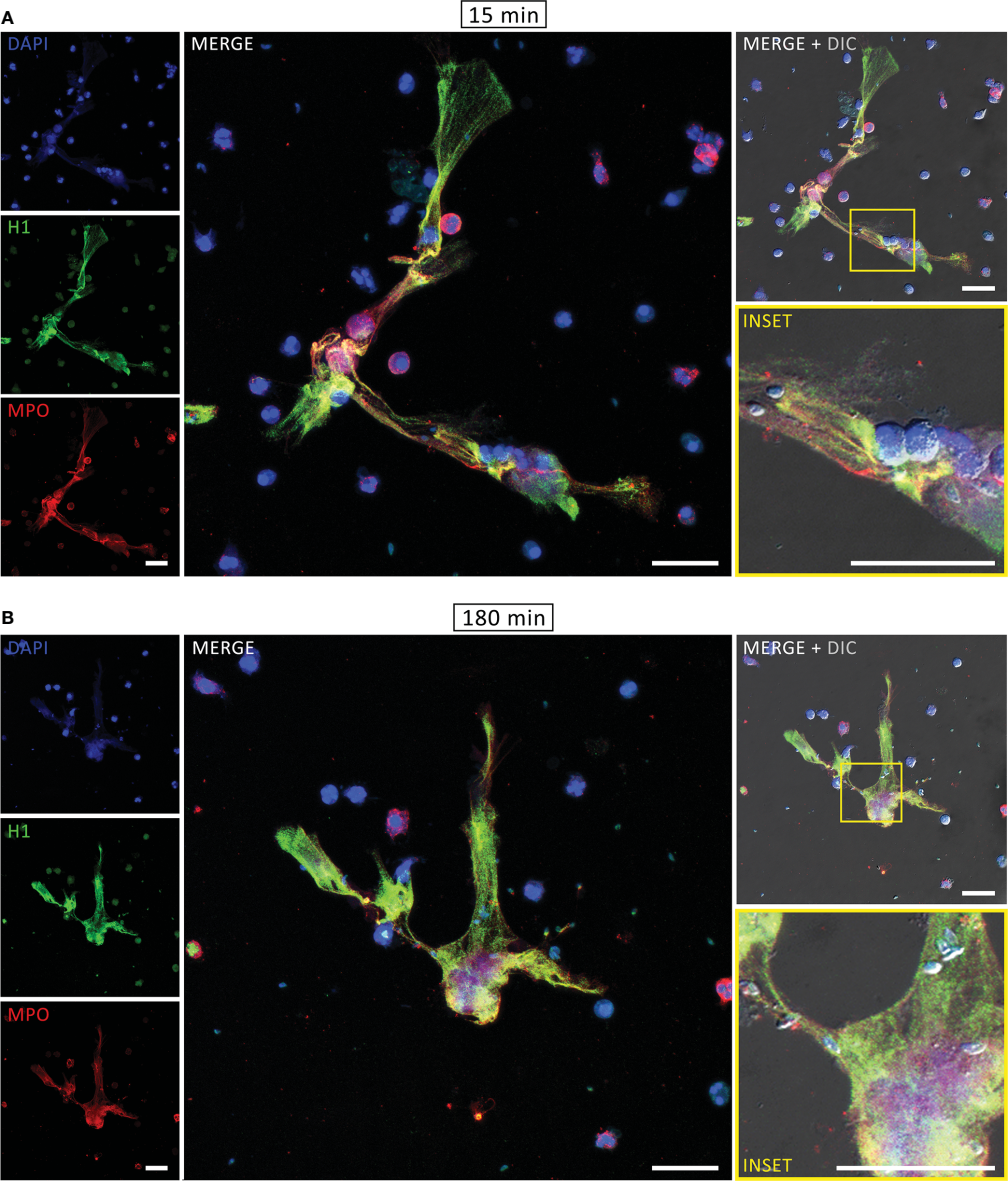
Rapid and classical NETs induced by *T. gondii* tachyzoites. Human neutrophils were incubated for 15 **(A)** or 180 min **(B)** with RH strain tachyzoites (5:1 parasites:neutrophil ratio), fixed and stained for histone H1 (*green*) and myeloperoxidase (MPO, *red*). DNA was counterstained with DAPI (*blue*). Bars = 30 µm.

**Figure 3:**
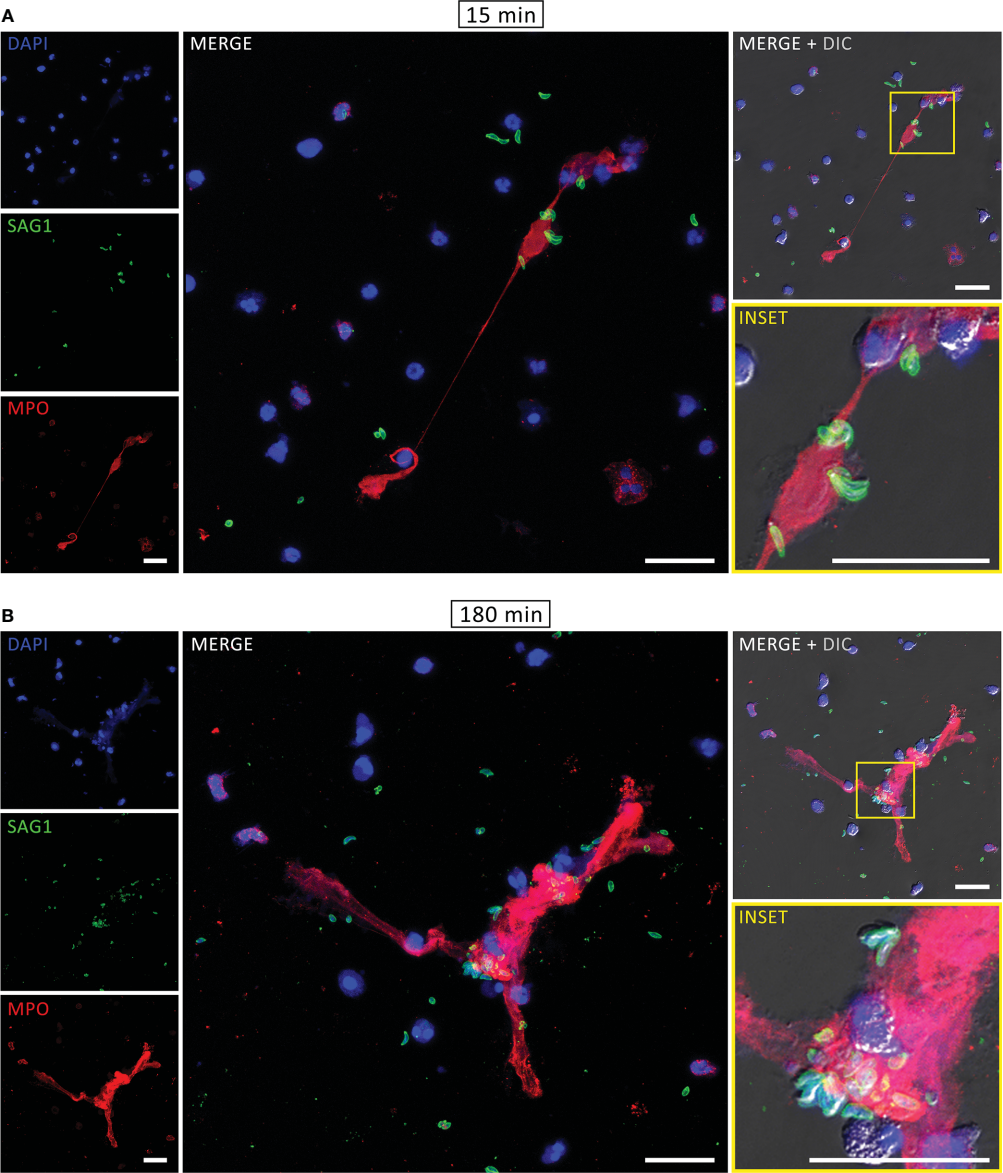
Rapid and classical NETs induced by *T. gondii* tachyzoites. Human neutrophils were incubated for 15 **(A)** or 180 min **(B)** with RH strain tachyzoites (5:1 parasites:neutrophil ratio), fixed and stained for myeloperoxidase (MPO, *red*) and SAG1 (p30, *green*). DNA was counterstained with DAPI (*blue*). Bars = 30 µm.

The components associated with human NETs were evaluated by immunofluorescence assay (IFA). After interaction with PMA for 180 min or tachyzoites for 15 or 180 min, neutrophils were fixed, stained for myeloperoxidase, histone H1 and DNA and examined under a confocal microscope. As expected, DNA structures associated with MPO and histone H1, which are some of the key signatures of NETs, were found after PMA treatment (**Supplementary Figure 2**). Similar staining was observed in both rapid and classical NETs, released in response to *T. gondii* (**Figure 2**). Furthermore, labeling the parasites with an antibody that recognizes *T. gondii* surface antigen 1 (SAG1) revealed tachyzoites associated with DNA-MPO structures at either early or late stages (**Figure 3**). Taken together, these data show that human neutrophils release classical and rapid NETs with typical components in response to tachyzoites of *T. gondii*.

### 3.2 NETs with multiple phenotypes are produced in response to *T. gondii* tachyzoites

**Figure 4:**
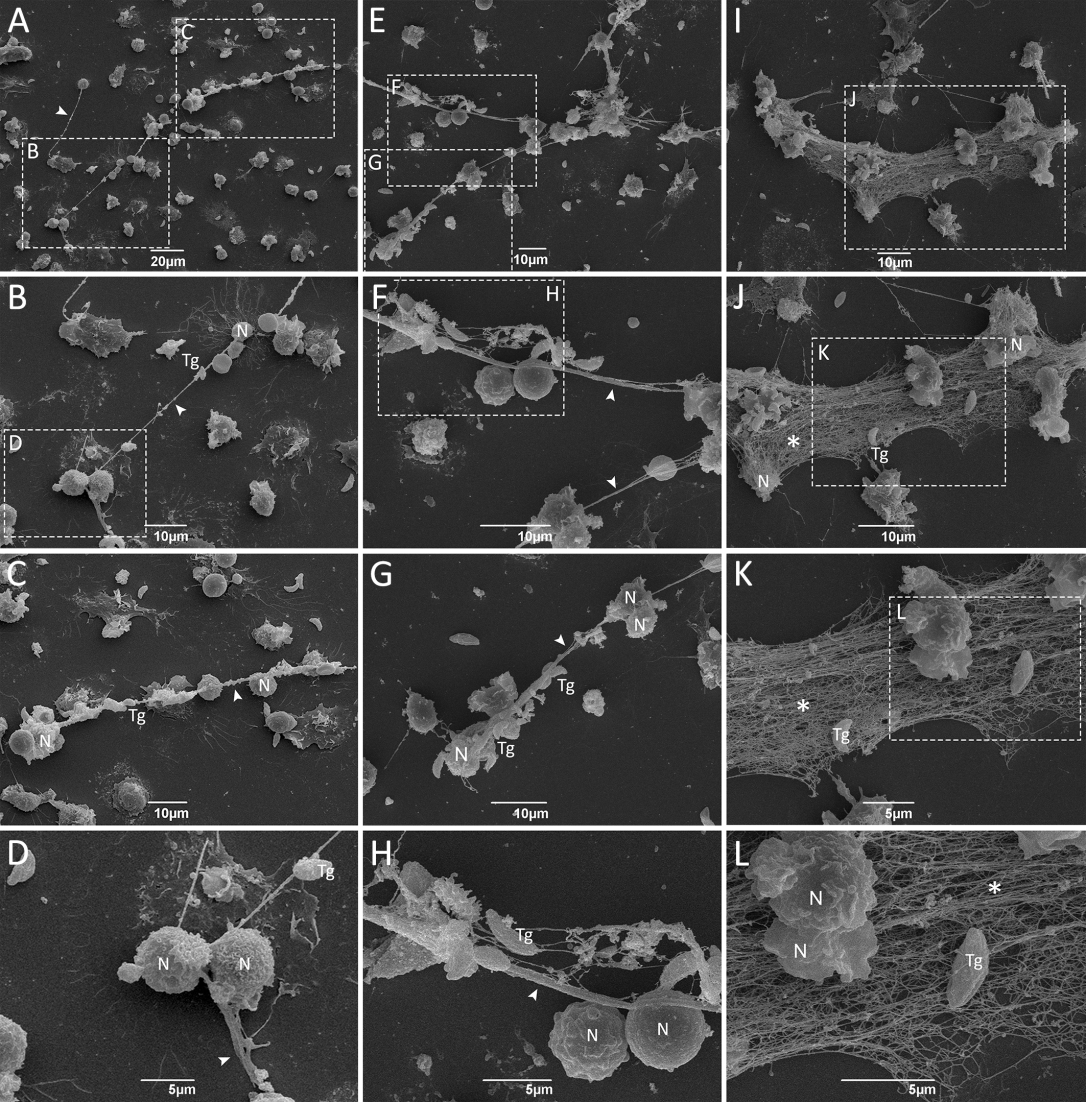
Ultrastructure of rapid and classical NETs induced by *T. gondii* tachyzoites as seen by SEM. Human neutrophils were incubated for 15 min **(A–H)** or 180 min **(I–L)** with RH strain tachyzoites (5:1 parasites:neutrophil ratio) and processed for SEM. Strands of chromatin fibers are indicated by arrowheads **(A–D, F–H)**, while spread NETs (*spr*NETs) are indicated by asterisks **(J–L)**. Tg, *T. gondii*; N, neutrophil.

We further investigated the ultrastructural aspects of classical and rapid NETs triggered by *T. gondii*. Neutrophils were incubated with tachyzoites for short or long times and processed for scanning electron microscopy (SEM) or transmission electron microscopy (TEM). By SEM we observed different types of traps following either classical or rapid stimulation (**Figure 4**, **Supplementary Figure 3**), including fine strands of DNA decorated with granules (**Figures 4A–H**), as well as intricated networks of DNA fibers, resembling spread NETs (*spr*NETs) (**Figures 4I–L**). Frequently, tachyzoites could be seen in association with neutrophil traps from all phenotypes. Of note, following rapid stimulation most of the neutrophils presented a healthy morphology, and it was not difficult to identify the cell from which NETs were originated (**Figures 4B–D**). On the other hand, classical production was often associated with neutrophils with unrecognizable morphology, possibly due to the death of cells, characteristic of the late release of NETs (**Figures 4I, J**). In this case, the cells from which the traps were released could not be determined.

**Figure 5:**
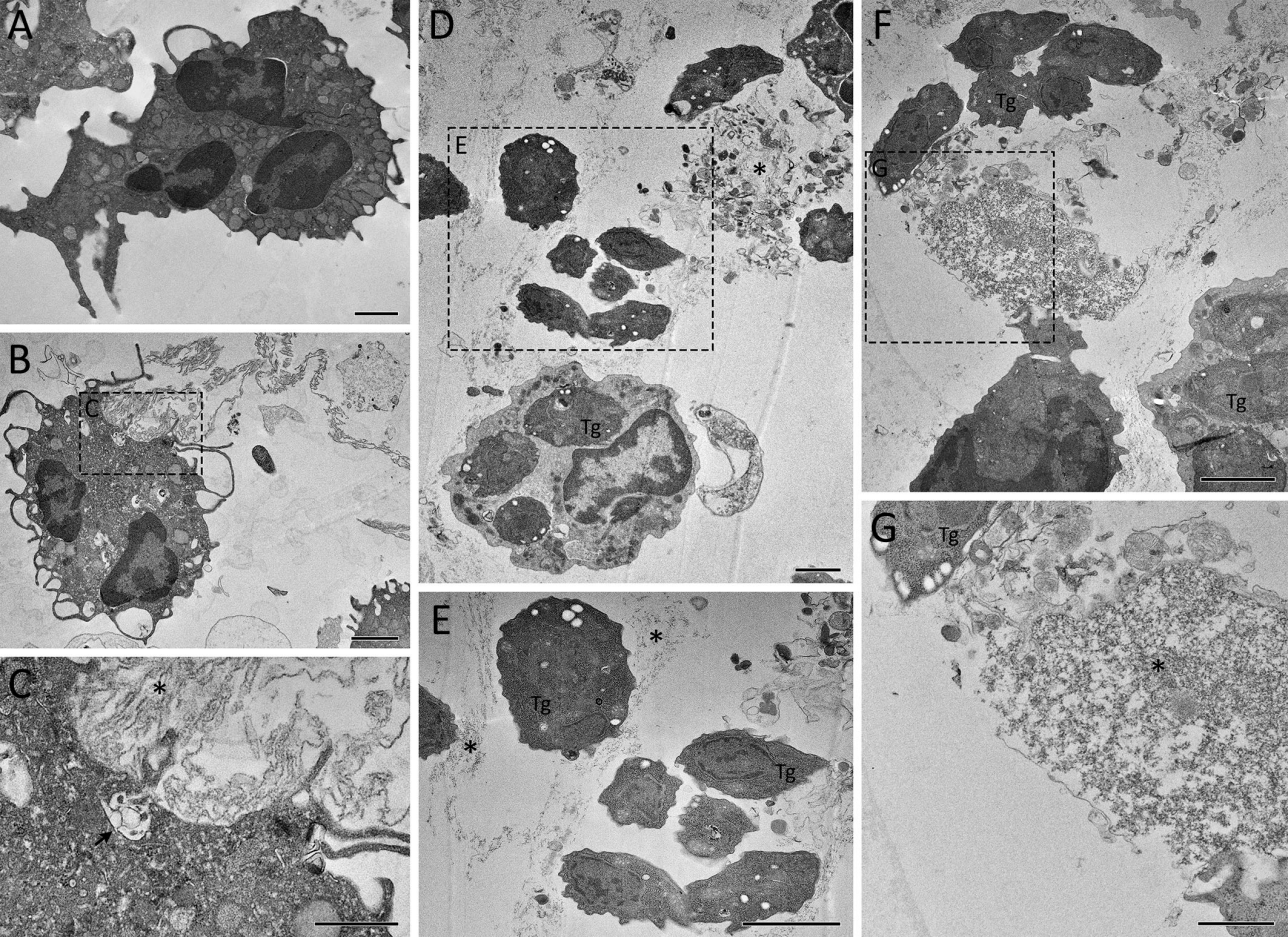
Ultrastructure of rapid and classical NETs induced by *T. gondii* tachyzoites as seen by TEM. Human neutrophils were incubated for 15 min **(B, C)** or 180 min **(D–G)** with RH strain tachyzoites (5:1 parasites:neutrophil ratio) and processed for TEM. An unstimulated neutrophil is seen in **(A)**. A vesicle discharging its content into the extracellular space is indicated by an arrow **(C)**, while NET-like structures are indicated by asterisks **(C–E, G)**. Tg, *T. gondii*. Bars = 1 µm **(A, B, D–F)** and 500 nm **(C, G)**.

Dramatic morphological changes in cell structure were observed after long periods of stimulation with parasites, as observated by TEM. Unstimulated neutrophils had a normal polymorphonuclear appearance, with intact membranes and contents (**Figure 5A**). Upon 15 min stimulation, neutrophils were seen to release DNA strand-like structures associated with granular content, consistent with NETs (**Figure 5B**). One can observe vesicles fused to the plasma membrane and discharging their contents into the extracellular environment (**Figure 5C**). After 180 min of interaction of neutrophils with *T. gondii*, NETs with different densities were frequently observed, associated or not with granules, and nearby parasites. Interestingly, parasites associated with NETs maintained their morphological integrity (**Figures 5D–G**).

**Figure 6:**
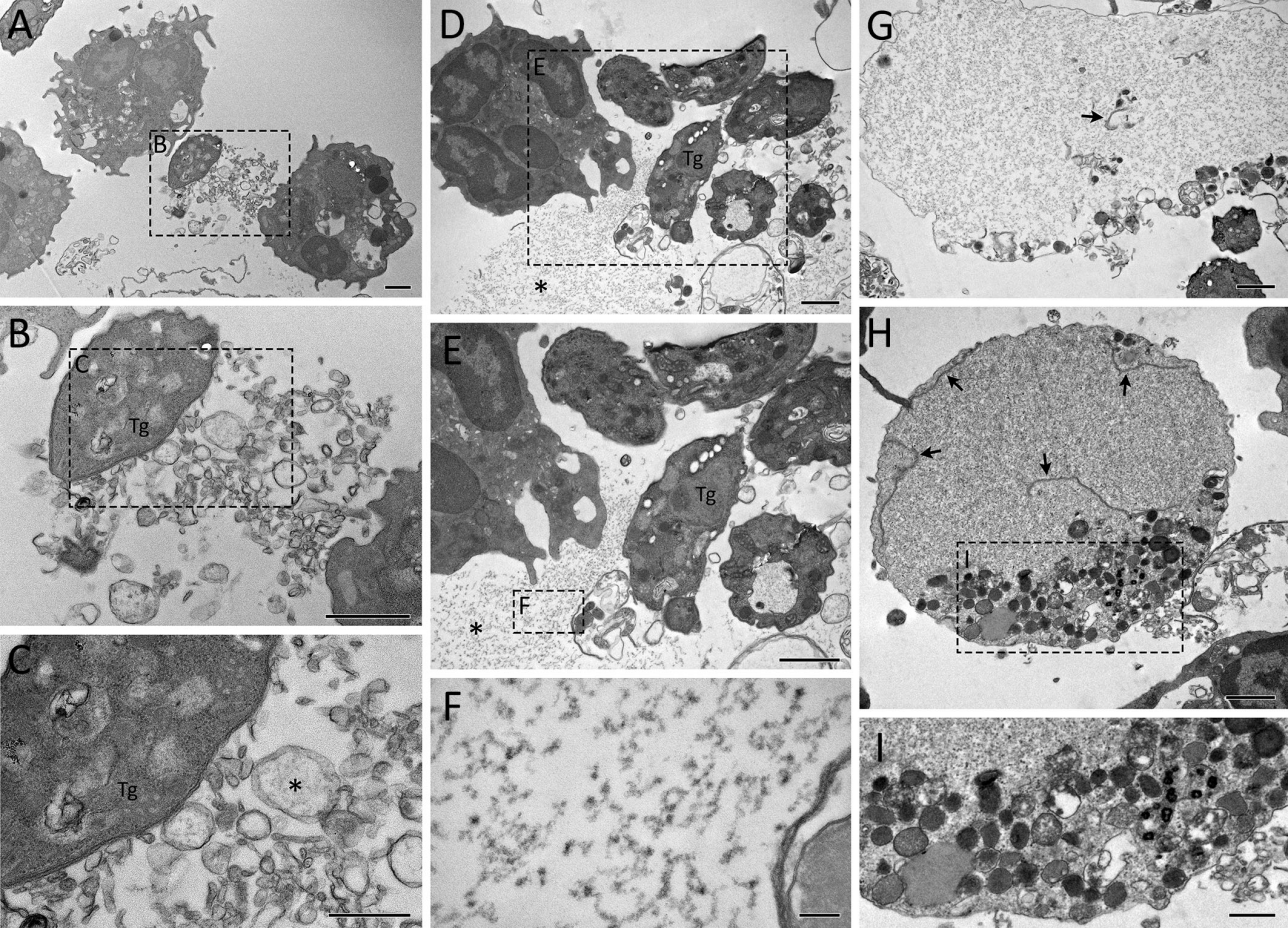
Ultrastructure of classical NETs induced by *T. gondii* tachyzoites as seen by TEM. Human neutrophils were incubated for 240 min with RH strain tachyzoites (5:1 parasites:neutrophil ratio) and processed for TEM. Remnants of the nuclear envelope are indicated by arrows **(G, H)**, while NET-like structures are indicated by asterisks **(C–E)**. Tg, *T. gondii*. Bars = 1 µm **(A, B, D, E, G, H)** and 100 nm **(C, F, I)**.

**Figure 7:**
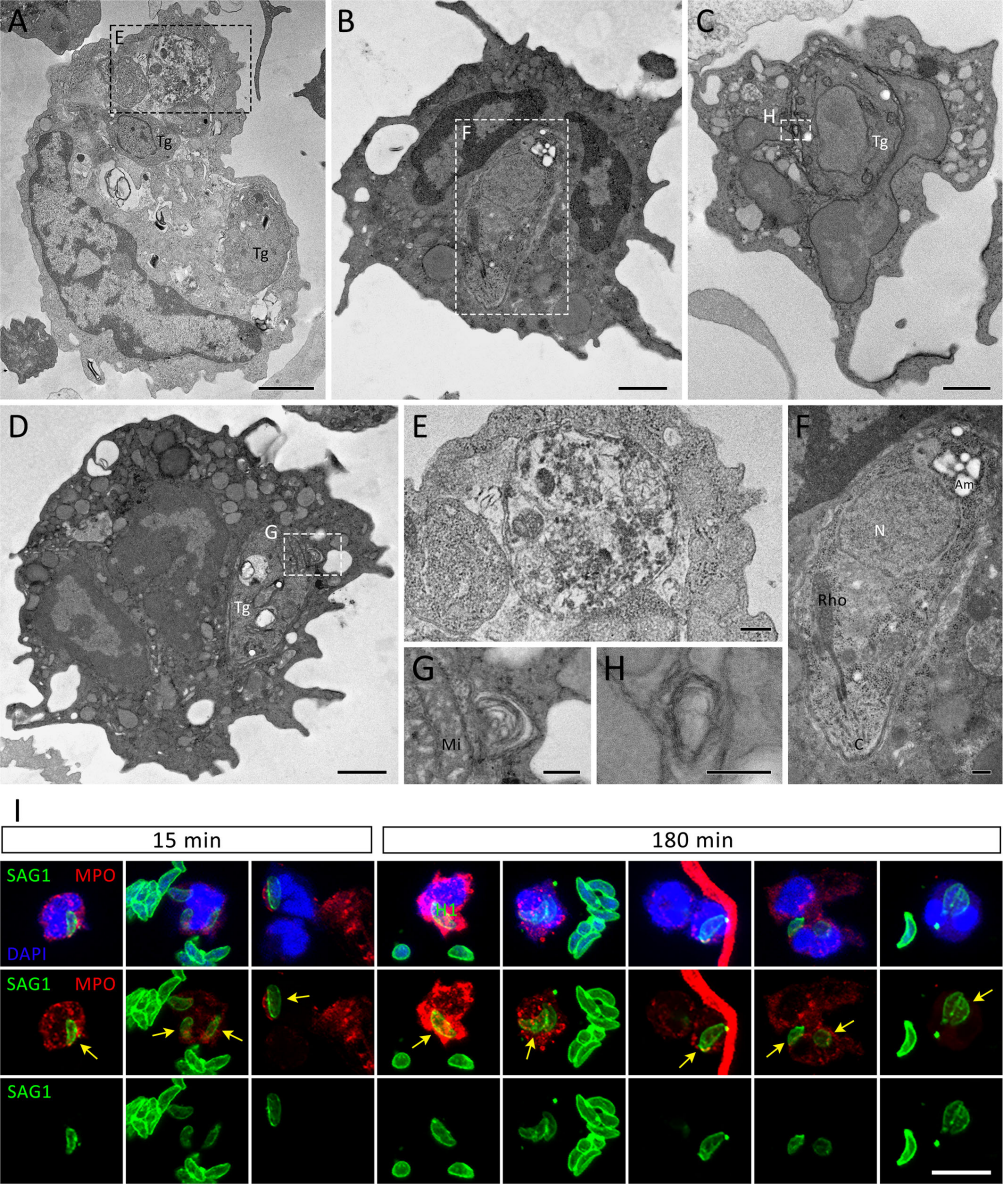
Internalization of *T. gondii* tachyzoites as seen by TEM and IFA. Human neutrophils were incubated for 15 min **(A, E, I)**, 180 min **(I)** or 240 min **(B–D, F–H)** with RH strain tachyzoites (5:1 parasites:neutrophil ratio) and processed for TEM **(A–H)** or stained for myeloperoxidase (MPO, *red*), SAG1 (p30, *green*), counterstained with DAPI (*blue*) and observed under a confocal microscopy **(I)**. The yellow arrows point to internalized parasites. Tg = *T. gondii*, Mi = Mitochondria, N = Nucleus, Rho = Rhoptries, Am = Amylopectin granules. Bars = 1 µm **(A–D)**, 0.2 µm **(E–H)** and 10 µm **(I)**.

After 240 min of interaction, cells with irregular shapes were commonly observed, as well as NET-like structures found free in the extracellular milieu or within membrane-bound entities (**Figure 6**). Tachyzoites were seen in association with free NETs (**Figures 6D, E**) as well as NET-containing vesicles (**Figures 6A–C**). NETs are recognized by their characteristic appearance resembling ‘beads on a string’ (**Figure 6F**), commonly reported as nucleosomes wrapped around DNA. Numerous anuclear neutrophils containing scattered chromatin and various cytoplasmic granules and vesicles were observed near the cell periphery, eventually fused with the plasma membrane (**Figures 6G–I**). The vestiges of the nuclear envelope are emphasized (arrows in **Figures 6G, H**). Not infrequently, neutrophils with internalized protozoa could also be found, either after short or long interaction periods (**Figures 5D, F**, **7A–H**), a finding that was confirmed by confocal microscopy (**Figure 7I**). In some cases, vacuoles containing what appear to be autophagosomes with parasites in process of degradation (**Figure 7E**) or myelin-like structures (**Figures 7G, H**), typical features of autophagy, could be observed at different time points.

Altogether, the ultrastructural investigations presented here indicate that human neutrophils can produce different types of NETs against *T. gondii* that entrap parasites. Furthermore, the internalization of tachyzoites occurs simultaneously with the release of NETs.

### 3.3 Production of NETs induced by *T. gondii* is concomitant to the release of H_2_O_2_ by neutrophils

**Figure 8:**
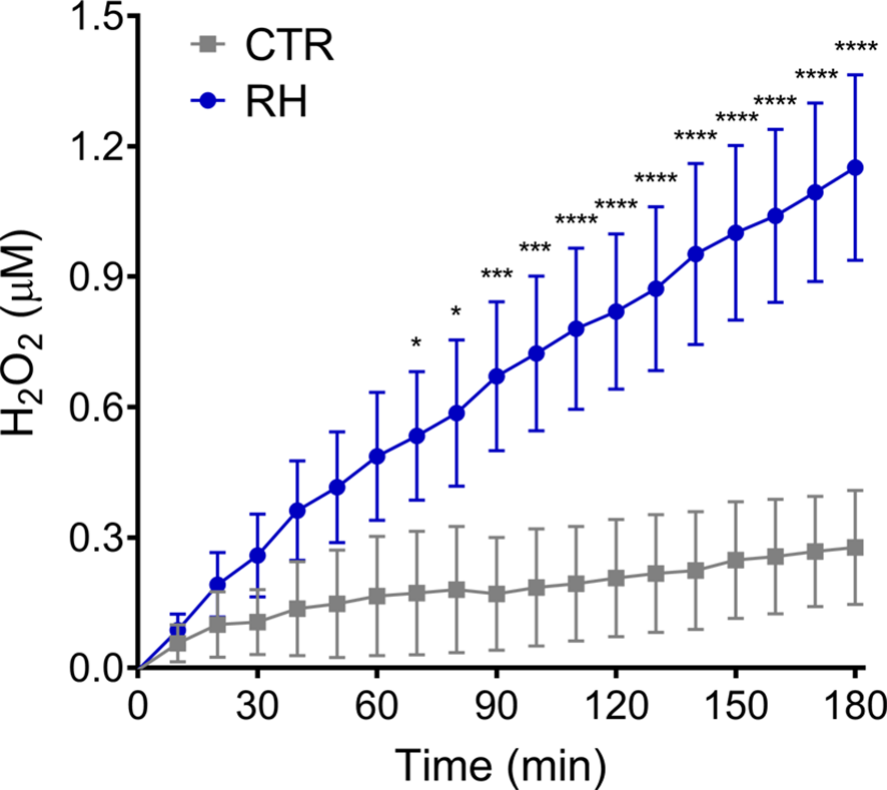
*T. gondii* induce the release of hydrogen peroxide by human neutrophils. Neutrophils from healthy donors were stimulated with tachyzoites from RH strain (5:1 parasites:neutrophil ratio) and the release of H_2_O_2_ by neutrophils was evaluated with Amplex Red Hydrogen Peroxide/Peroxidase Assay Kit. Results are shown as means (SD) (*n* = 3). * *p* < 0.05; *** *p* < 0.001; **** *p* < 0.0001.

To investigate the involvement of reactive oxygen species (ROS) during NET production, neutrophils were exposed to *T. gondii* tachyzoites and hydrogen peroxide release was evaluated over time by the Amplex Red assay. We observed that neutrophils exposed to parasites continuously release H_2_O_2_ as a function of time (**Figure 8**), indicating that human neutrophils are activated by *T. gondii* to release ROS concomitantly with the release of NETs.

### 3.4 Inhibition of NE, PAD, calcium and PI3K decreases the release of human NETs

**Figure 9:**
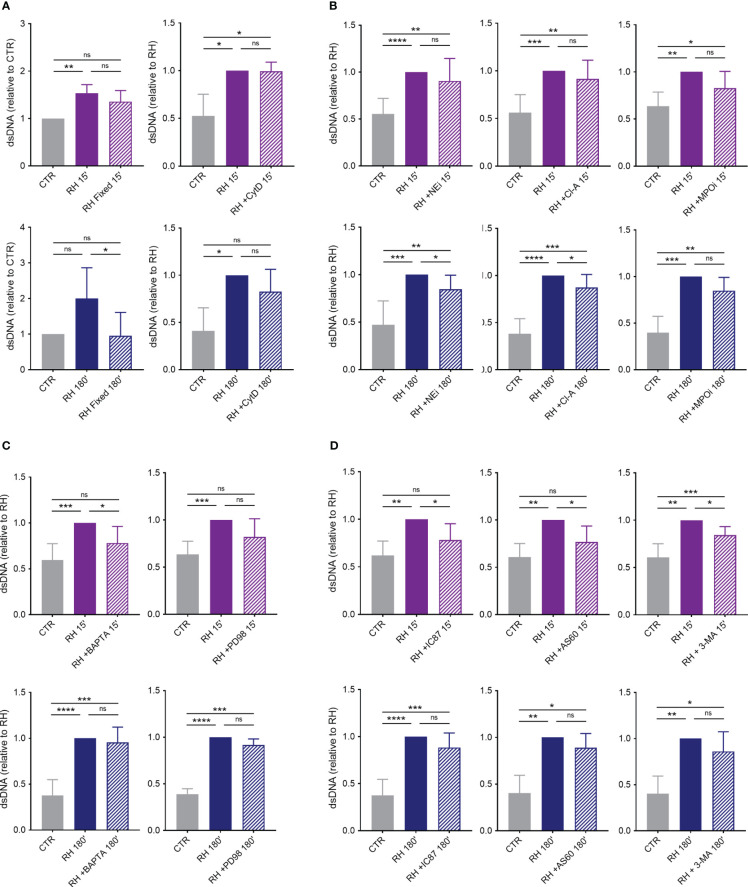
Mechanisms behind human NETs release induced by *T. gondii*. **(A)** Human neutrophils were incubated for 15 or 180 min in the absence (CTR) or presence of alive (RH) or fixed tachyzoites (RH Fixed) or were pretreated or not with cytochalasin D (CytD; 10 μg/mL) for 30 min and then stimulated with *T. gondii* tachyzoites (5:1 parasites:neutrophil ratio) (*n* = 5). **(B)** Neutrophils were pretreated or not with the neutrophil elastase inhibitor MeOSuc-AAPV-CMK (NEi; 10 μg/mL), the PAD inhibitor chloroamidine (Cl-A; 12 μM), or the myeloperoxidase inhibitor I (MPOi; 600 nM) for 30 min and then stimulated for 15 or 180 min with RH tachyzoites (*n* = 7–11). **(C)** Cells were pretreated or not with the intracellular calcium chelator BAPTA/AM (BAPTA; 10 μM) or the MEK inhibitor PD98059 (PD98; 60 µM) for 30 min before stimulation with parasites (*n* = 8). **(D)** Neutrophils were incubated of not with the PI3Kδ selective inhibitor IC87114 (IC87; 1 μM), the PI3Kγ selective inhibitor AS605240 (AS60; 10 µM), or the class III PI3K Vps34 and autophagy inhibitor 3-methyladenine (3-MA; 5 mM) for 30 min before interaction with parasites (*n* = 6–9). All supernatants were collected and released dsDNA was quantified with PicoGreen Kit. Results are shown as means (SD). * *p* < 0.05; ** *p* < 0.01; *** *p* < 0.001; **** *p* < 0.0001.

The mechanisms behind NETs production induced by *T. gondii* were further investigated. Our initial inquiry focused on ascertaining if the internalization of tachyzoites by human neutrophils is a prerequisite for initiating the release of extracellular traps. To do this, we initially compared neutrophil response to either alive or paraformaldehyde-fixed tachyzoites. In contrast to alive parasites, in our experimental conditions fixed parasites were not able to induce NETs release (**Figure 9A**). Subsequently, we exposed neutrophils to the actin polymerization inhibitor cytochalasin D prior to the incubation of neutrophils with parasites. By doing so, we prevented the internalization of parasites by phagocytosis. Cytochalasin D treatment did not affect classical or rapid NET production (**Figure 9A**).

To further elucidate the role of molecules associated with the decondensation of chromatin during NET release, we pretreated human neutrophils with inhibitors of NE, PAD and MPO before adding parasites. Administration of NE and PAD inhibitors led to a decrease in the release of classical but not rapid NETs (**Figure 9B**). On the other hand, MPO inhibition did not significantly affect dsDNA release by neutrophils stimulated for either 15 or 180 min with *T. gondii* (**Figure 9B**).

We next examined the participation of calcium and the MAPK/ERK signaling pathway in NETs triggered by tachyzoites. Pretreatment of cells with a calcium chelator led to significant inhibition of rapid but not classical release of traps by human neutrophils (**Figure 9C**). However, inhibition of MEK 1/2 had no significant effect on dsDNA release by human cells challenged for either 15 or 180 min with *T. gondii* (**Figure 9C**).

Finally, we investigated the role of PI3K signaling pathway in NETs induced by *T. gondii* and found that inhibition of PI3Kδ or PI3Kγ partially prevented dsDNA release by human neutrophils stimulated with parasite for 15, but not for 180 min (**Figure 9D**). The same result was observed when we pretreated cells with 3-MA, a class III PI3K Vsp34 and autophagy inhibitor (**Figure 9D**). It is noteworthy that none of the inhibitors used here showed toxicity for neutrophils after 180 min of incubation (**Supplementary Figure 4**).

### 3.5 Human NETs do not affect the viability and infectivity of *T. gondii* tachyzoites

**Figure 10:**
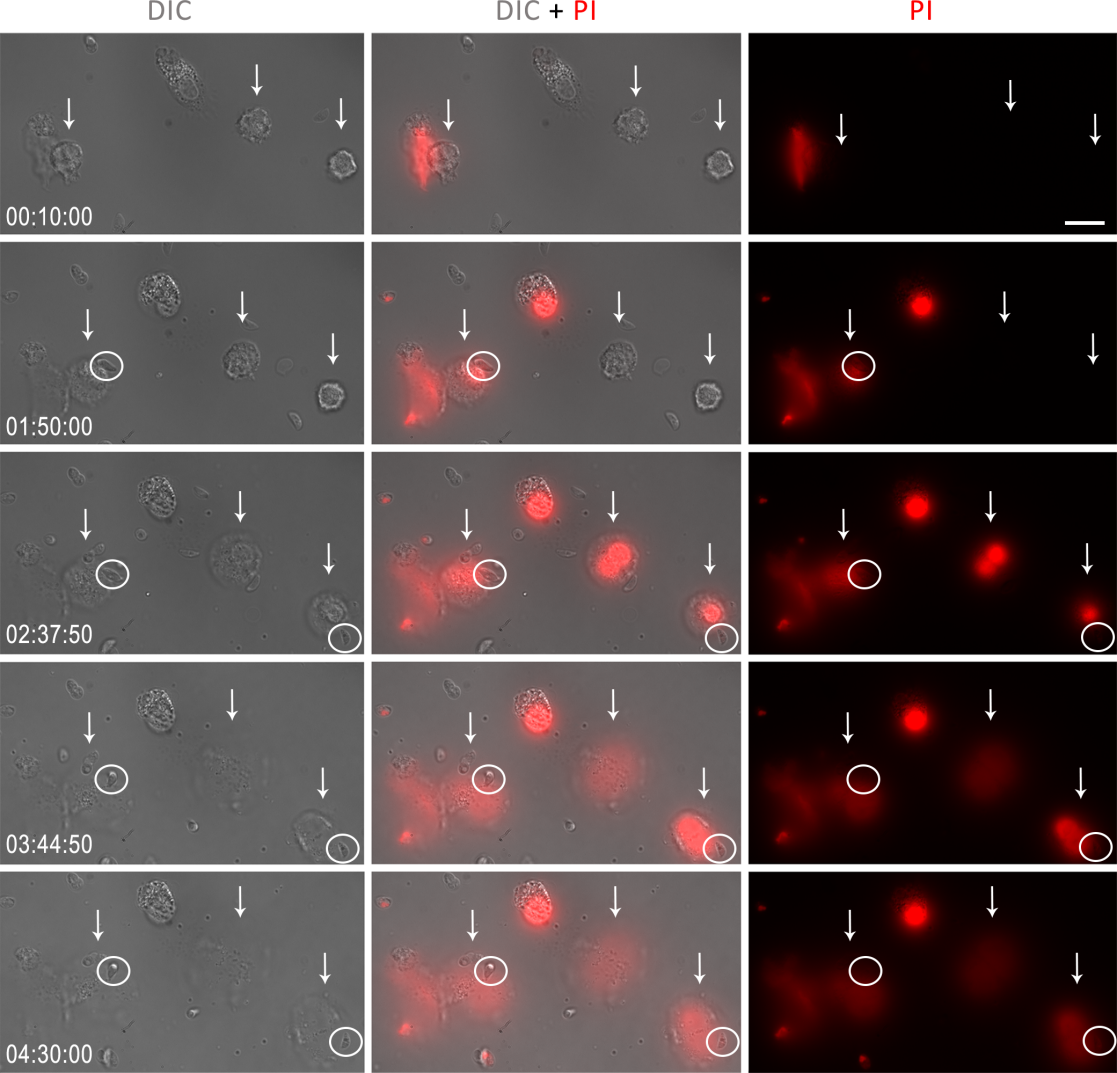
Human neutrophils were stimulated with *T. gondii* tachyzoites of the RH strain (5:1 parasites:neutrophil ratio) and incubated with PI (3 μg/ml). The times in the lower left corner indicate, from 10 min of interaction until the end of the recording, the moment of NET release by neutrophils (white arrows). The white circles indicate tachyzoites in contact with NET. Bar = 20 µm.

To investigate the ability of human NETs to affect parasite viability, we performed a time-lapse analysis of neutrophils with *T. gondii* in the presence of the DNA intercalator propidium iodide (PI). As expected, in the presence of the parasite, neutrophils have their morphology altered over time, incorporating PI – an indication of impaired membrane integrity –, and ending with the extrusion of NETs, which is marked by the expansion of the surrounding chromatin around the neutrophils (**Figure 10**). Surprisingly, parasites associated with human NETs did not incorporate PI for up to 270 min of interaction.

At the end of the experiments, some dead tachyzoites stained for PI were observed, but these were not related to the association of parasites with neutrophils or NETs (**Supplementary Figure 5**). The viability of *T. gondii* over time was confirmed by comparing tachyzoites kept alone under the same experimental conditions with parasites kept in an ice bath for 270 min. There was no significant spontaneous death of tachyzoites during the entire observation period (**Supplementary Figure 6**).

In view of a more detailed study of the release of NETs in response to *T. gondii* and bearing in mind that contact with traps does not seem to affect the viability of the parasite, we questioned whether NETs would lead to entrapment and, consequently, a decrease in the infection rate of the host cell. To address this issue, we treated tachyzoites with 1% RPMI control medium (CTR), NET-conditioned medium obtained from stimulated neutrophils (CM NET), NET-conditioned medium after filtration to retain the traps (CM NET FL) or conditioned medium without stimulus obtained from unstimulated neutrophils (CM CTR) for subsequent infection of VERO cell monolayers.

**Figure 11:**
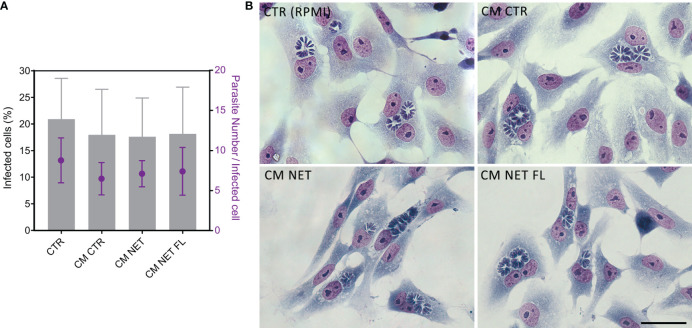
Infection of Vero cells with NET-treated tachyzoites. **(A)** *T. gondii* tachyzoites from the RH strain were treated with culture medium alone CTR (RPMI), conditioned medium from unstimulated neutrophils (CM CTR), NET-conditioned medium from stimulated neutrophils (CM NET) or NET-conditioned medium after filtration to retain the traps (CM NET FL), and used to infect a monolayer of VERO cells (1:1 parasites:Vero ratio). After 24 h of interaction, samples were processed, stained using the May Grunwald Giemsa method, and analyzed. **(B)** Representative images of coverslips infected at each condition and stained with May Grunwald Giemsa. *n* = 3–7.

The amount of dsDNA from CM NET was 4.8 times higher than that from CM CTR (mean (SD) range of 739 ± 468 and 153 ± 58 ng/mL for CM NET and CM CTR, respectively, * *p* < 0.05, *n* = 3). In another set of experiments, the amount of dsDNA increased from 214 ± 142 (CM CTR) to 552 ± 62 (CM NET), dropping to 294 ± 99 ng/mL after filtration (CM NET FL) (CM CTR x CM NET, * *p* < 0.05; CM NET x CM NET FL, ** *p* < 0.01; CM CTR x CM NET FL, ns, *n* = 4). After the infectivity test, we quantified the infected cells and the amount of parasites per cell. Our results indicate that there was no significant difference between the groups, either in the proportion of infected cells or in the quantity of parasites within each cell (**Figure 11**). In addition, parasites alone cultured in CM NET remained viable for up to 270 min of interaction (**Supplementary Figure 6**).

Together, these results indicate that, although human NETs are produced against *T. gondii* tachyzoites and entrap the parasites, they do not significantly affect protozoan viability and its ability to infect the host cell.

## 4 Discussion

The investigation into the relevance of NETs within the context of *T. gondii* infection has garnered our attention as well as that of fellow researchers over the past decade. While the production of traps by neutrophils from different hosts has been established, the specific role of human NET release as a defense mechanism against the parasite remains to be fully elucidated. Hence, the objective of this study was to explore the human neutrophils’ response to tachyzoites of the highly virulent RH strain of *T. gondii*, encompassing the unraveling of the underlying mechanisms of NET release and the understanding of its significance in the context of infection control.

We demonstrated here that human neutrophils release rapid NETs in response to *T. gondii* tachyzoites. These newly identified traps, in addition to the classical traps previously described, were detected as early as 15 min post-stimulation. This observation is in line with previous studies conducted on neutrophils from other *T. gondii* hosts, both intermediate and definitive, as reported by Reichel et al. (26), Yildiz et al. (27, 28), Macedo et al. (32), and Simsek et al. (34). Through immunofluorescence analysis, we established that both forms of NETs are primarily composed of histones and MPO and contribute to parasite entrapment. Ultrastructural studies revealed the intricate interaction between traps and protozoan, showcasing diverse phenotypes. Prior studies identified distinct phenotypes based on structural morphology, including spread NETs (*spr*NETs), diffuse NETs (*diff*NETs), and aggregated NETs (*agg*NETs), each with unique characteristics (38, 39). Here we demonstrate that human NETs triggered by *T. gondii* consist of fine strands of DNA, characterized by elongated thread-like structures located near intact cells, or elongated and thin web-like structures of decondensed chromatin, resembling *spr*NETs. Our findings are consistent with our prior research involving cat neutrophils (32) and align with observations made by Imlau et al. (30) in their study of dolphin NETs generated against the pathogen. However, in contrast to dolphins, where large clusters of NETs aggregating neutrophils, known as *agg*NETs, were also frequently present, here we could only find small clusters of NETs with neutrophils.

We further aimed to assess how the parasite activates human neutrophils for NET production. Abi Abdallah’s (25) study demonstrated that cytochalasin D had no impact on classical traps generation in human neutrophils. Our study corroborated and extended these findings, showing that rapid NETs are also unaffected by CytD treatment. This is consistent with our previous work on feline neutrophils (32), emphasizing NET release’s autonomy from phagocytosis in *T. gondii* intermediate and definitive hosts. In line with these results, Miranda et al. (37) demonstrated that treating tachyzoites with mycalolide B, an irreversible inhibitor of actin polymerization, completely suppressed parasite entry and NET production. Our study further confirmed that stimulating neutrophils with deceased microorganisms did not induce NETs, emphasizing the requirement for parasite viability to trigger cellular activation and subsequent NET generation. Although we cannot rule out the potential contribution of soluble factors released by *T. gondii* in NET induction, the data presented here, along with other studies, suggest that the internalization of *T. gondii* through active invasion, rather than phagocytosis, triggers the release of both rapid and classical NETs. In our preparations, we indeed observed numerous neutrophils containing internalized tachyzoites at both interaction time points, confirming previous findings that demonstrated a high infection efficiency of neutrophils by the same strain of *T. gondii* (40). Since parasites from different genetic backgrounds enter cells through distinct mechanisms (41), the results obtained with a less virulent strain of *T. gondii* may differ from the results we obtained here.

From cellular activation to NET release, various molecular mechanisms fully or partially participate. In our study, we investigated the pathways involved in the formation of human traps, comparing rapid and classical NET release, examining the production of ROS by neutrophils, and conducting pharmacological inhibitor assays. For the latter, NETs release was estimated by the quantification of extracellular DNA using the PicoGreen reagent. Although this method has limitations as it provides no information about how the cells died (42), we demonstrated through various microscopy techniques that *T. gondii* infection indeed triggers the release of NETs. Importantly, previous research has shown that *T. gondii* infection prolongs neutrophil survival by inhibiting apoptosis, which promotes the maintenance of a replicative niche for the parasite (43). Moreover, we confirmed that none of the inhibitors employed in our study exhibited any cytotoxicity towards neutrophils after 180 minutes of incubation. Thus, the data observed with PicoGreen here are likely attributed to the release of NETs, rather than other forms of cell death.

We initially evaluated simultaneous ROS production and NET generation, observing increased H_2_O_2_ production over time upon *T. gondii* stimulation. Our data corroborate others, which demonstrated that NADPH oxidase inhibition reduced the classic NET production by neutrophils from harbour seals (26) and dogs (33), or that human neutrophils produce ROS against the parasite (37).

We then assessed a crucial step in NET release: chromatin decondensation. This may involve NE and MPO translocating into the nucleus (44), along with PAD_4_ catalyzing histone hypercitrullination (45). In contrast to Reichel et al. (26), who identified the dependence of MPO and NE on the release of harbor seals NETs against *T. gondii*, our model showed the presence of MPO in the NET structure without altering rapid or classical NET production upon its inhibition. Conversely, NE inhibition reduced classical NET production, consistent with the findings of Miranda et al. (37), highlighting the role of NE in human traps production. Similar outcomes were observed with cat neutrophils (32). Furthermore, Cl-amidine, a PAD inhibitor, reduced classical production without affecting rapid NETs, underscoring this enzyme’s primary role in classical human NET formation in response to *T. gondii*.

Regarding the signaling pathways involved in human NET generation against *T. gondii*, we demonstrated for the first time that treating neutrophils with BAPTA, an intracellular calcium chelator, significantly reduced rapid traps release. We and others have previously shown calcium’s role in NET formation by neutrophils from different hosts, though those analyses were conducted only with extended inductions, where blocking Ca^2+^ signaling negatively affected classical release (26, 32). Abi Abdallah and colleagues (25) revealed NET production leads to ERK1/2 activation and partially relies on the MEK1/2 signaling pathway. They found that inhibiting this pathway with U0126 reduces NET production. In our study, we used the PD98 inhibitor, which also targets the MAPK/ERK pathway. However, to our surprise, this didn’t alter rapid or classical release. While both inhibitors specifically target MEK1/2, PD98 might not inhibit MEK2 as effectively as U0126 (46). As a result, we cannot exclude the MEK pathway’s involvement in human NET release in response to the protozoan, and further experiments with different inhibitors are required for a more comprehensive study of this signaling pathway.

The PI3K pathway is expressed in human neutrophils and regulates various immune cell functions, including ROS production (47). We previously revealed that the classical traps production induced by *Leishmania amazonensis* partly depends on PI3K, mediated by PI3Kγ and PI3Kδ isoforms (48). Moreover, our group demonstrated the involvement of the PI3K pathway in classical NET release by cat neutrophils (32). Here, we evidenced the participation of both PI3Kγ and PI3Kδ isoforms in rapid NET release, thereby establishing the first link of this pathway to human neutrophil NET production against *T. gondii*. Notably, pretreatment of cells with 3-MA also partially prevented NET release. 3-MA is known to inhibit autophagy by blocking autophagosome formation through the inhibition of the class III PI3K complex (49). Autophagy is a physiological process of self-digestion of nonfunctional organelles and/or macromolecules, which maintains eukaryotic homeostasis (50). It is also essential for several neutrophil functions, including phagocytosis, degranulation, and the release of NETs (51). Recent evidence indicates that autophagy either occurs concurrently with or primes neutrophils to trigger NETs (52, 53). *Besnoitia besnoiti*, an apicomplexan protozoan closely related to *T. gondii*, was shown to induce NET formation and autophagy simultaneously in bovine neutrophils (54). Our results with 3-MA, together with our TEM findings of autophagosome- and myelin-like structures in neutrophils stimulated with *T. gondii* tachyzoites, indicate that autophagy occurs concomitantly with, and might play a role in NET release, especially in the case of rapid NETs, triggered by the parasite. Further investigations are warranted to explore this issue in detail.

The structure of NETs and their antimicrobial components enable the entrapment and destruction of various pathogens. Our studies, as well as others, consistently show that neutrophils from cats, cattle, donkeys, harbour seals, mice, and sheep all produce NETs that inhibit *T. gondii* infectivity or impact parasite viability (25–28, 32). Surprisingly, we reveal that this does not apply to human traps. Neither parasite viability nor infectivity to host cells was affected by human NETs. This phenomenon can be interpreted from different angles.

Neutrophils from various species exhibit distinct activities like phagocytosis, activation status, ROS production, and pathogen killing. For instance, avian heterophils, akin to mammalian neutrophils and recently identified as NET producers against *T. gondii* (35), display reduced phagocytosis, lower bactericidal activity, and diminished oxidant production in response to zymosan compared to dog and human neutrophils (55). Conversely, human neutrophils generate considerably more H_2_O_2_ and experience more elevation in intracellular Ca^2+^ levels than canine neutrophils or chicken heterophils when exposed to the chemotactic peptide known as N-formyl-methionyl-leucyl-phenylalanine (fMLP) (55). In this study, we observed several neutrophils that had internalized parasites, with some of them in the process of degradation. This indicates the occurrence of an active phagocytic process. As the production of NETs coincides with phagocytosis, their potential role in trapping tachyzoites for subsequent engulfment by human neutrophils cannot be ruled out.

The signaling pathways, granule protein content, receptor expression, and repertoire of secreted molecules also diverge between mouse and human neutrophils, as recently summarized by Nauseef (56). For instance, unlike mouse neutrophils, human neutrophils do not produce IFN-β, IL-10, or IL-17 (56), all crucial factors in protecting against *T. gondii* in murine infection models (57–62). Consequently, given the myriad effects of cytokines and other molecules produced by neutrophils, the immune response would significantly vary across distinct host species from which cells were isolated. This diversity makes it challenging to predict outcomes, particularly in *in vitro* settings where other contributing factors are absent.

Numerous agents have been demonstrated to induce the generation of NETs (63), yet the complete elucidation of the molecular pathways underlying this phenomenon remains a distant goal (64). In comparative proteomic analyses, researchers have shown that human neutrophils obtained from healthy donors (65) or from patients with rheumatoid arthritis or systemic lupus erythematosus (66) produce heterogeneous NETs, with variations in protein composition and post-translational modifications, triggered by diverse stimuli. Further investigation is warranted to determine the extent to which neutrophils from different host species produce NETs with varying contents in response to a singular stimulus, such as *T. gondii* tachyzoites, leading to distinct outcomes in parasite control.

Finally, it is crucial to bear in mind that different microorganisms have developed strategies to evade NETs and sustain infections, such as degrading NETs with nucleases, resisting their effects via biofilms, capsules, or surface modifications, and hindering their microbicidal effects with degrading molecules (67). In a prior investigation, we demonstrated that *L. amazonensis* produces a 3’-nucleotidase/nuclease that enables promastigotes to evade NET-mediated killing by degrading the DNA backbone of NETs (68). In a study conducted by Wei and colleagues (33), canine neutrophils were utilized to investigate the interplay between *T. gondii* tachyzoites and zymosan-induced NETs. This inquiry unveiled the degradation of NETs when parasites were present. Whether *T. gondii* tachyzoites employ nucleases similar to those seen in *L. amazonensis*, or utilize other mechanisms to evade NETs, remains an unanswered question that merits deeper investigation.

**Figure 12:**
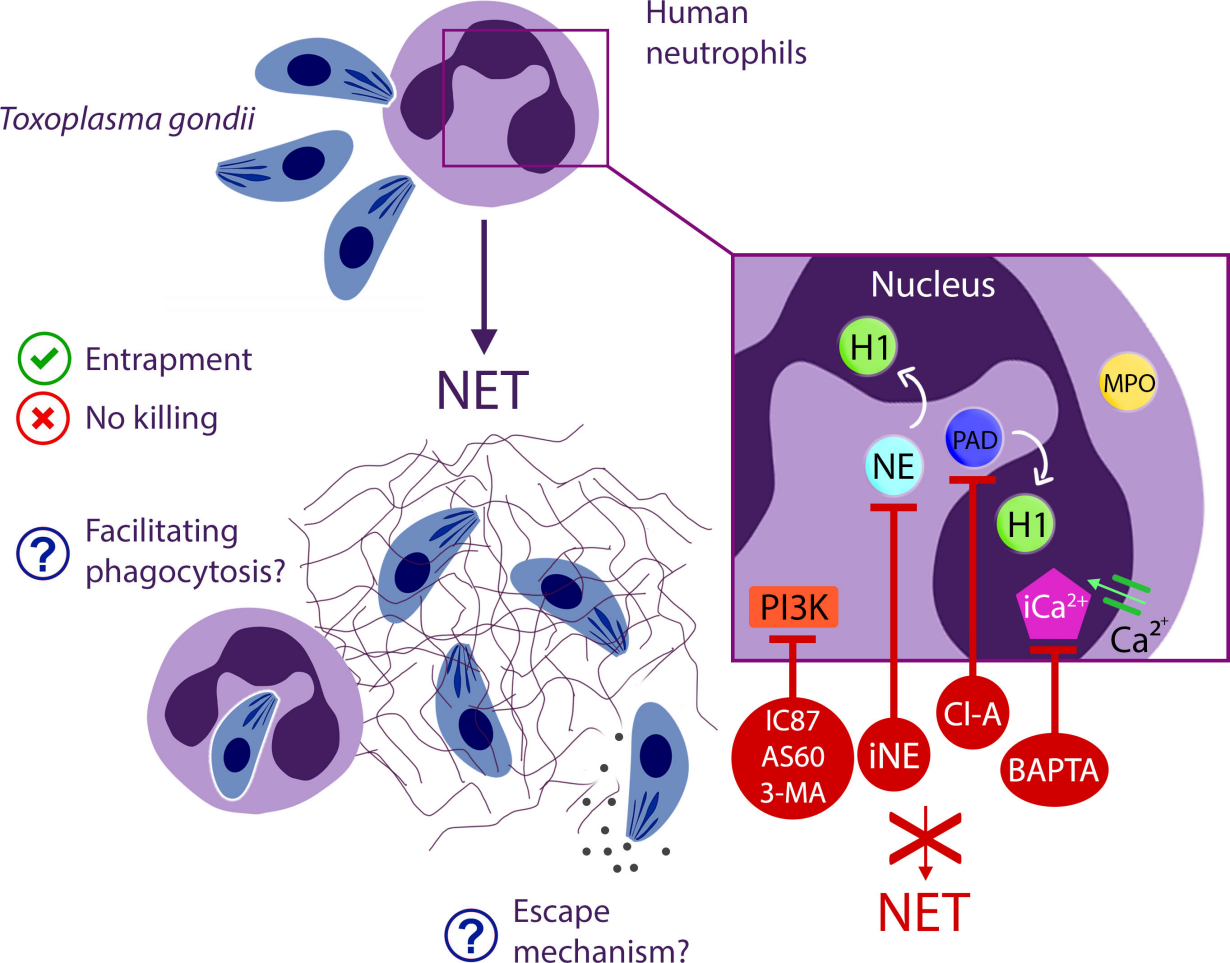
Comprehensive insight into NET production events elicited by human neutrophils in response to *T. gondii*.

In conclusion, we have demonstrated that human neutrophils release rapid NETs in addition to the classical NETs triggered by *T. gondii* tachyzoites. The structure of the traps exhibited different phenotypes over the analyzed time periods while maintaining an association with the parasites. The mechanisms driving NETs generation include NE and PAD and involves intracellular Ca^2+^ and the PI3K signaling pathway. Surprisingly, human traps alone do not impair parasite viability or infectivity. However, they may assist neutrophils in entrapping parasites for subsequent phagocytosis and elimination (**Figure 12**). This investigation sheds light on a new dimension in the host’s battle against *T. gondii*, contributing to our broader understanding of the complex interaction between pathogens and the human immune response.

## Data availability statement

The raw data supporting the conclusions of this article will be made available by the authors, without undue reservation.

## Ethics statement

All procedures involving human cells were performed with blood samples obtained after written informed consent and in full compliance with the guidelines of the Research Ethics Committee of the Hospital Universitário Clementino Fraga Filho – UFRJ and approved under number CAAE 49889621.7.0000.5257. Additionally, the experimental procedures involving mice were carried out in strict compliance with the recommendations of the Brazilian National Council for the Control of Animal Experimentation (CONCEA). All procedures were approved by the Ethics Committee for the Use of Animals from the Instituto Oswaldo Cruz - Fiocruz (CEUA/IOC, license number L-042/2018-A2).

## Author contributions

IM: Conceptualization, Data curation, Investigation, Methodology, Visualization, Writing – original draft, Writing – review & editing. FL: Investigation, Resources, Writing – review & editing. HB: Resources, Writing – review & editing. ES: Resources, Writing – review & editing. RM-B: Resources, Writing – review & editing. RM: Conceptualization, Data curation, Formal Analysis, Funding acquisition, Investigation, Methodology, Project administration, Resources, Supervision, Validation, Visualization, Writing – original draft, Writing – review & editing.

## References

[1] Smith NC Goulart C Hayward JA Kupz A Miller CM van Dooren GG. Control of human toxoplasmosis. Int J Parasitol (2021) 51(2-3):95–121. doi: 10.1016/j.ijpara.2020.11.001 33347832

[2] Dunay IR Gajurel K Dhakal R Liesenfeld O Montoya JG. Treatment of toxoplasmosis: historical perspective, animal models, and current clinical practice. Clin Microbiol Rev (2018) 31(4):e00057–17. doi: 10.1128/CMR.00057-17 PMC6148195 30209035

[3] Wang ZD Liu HH Ma ZX Ma HY Li ZY Yang ZB. *Toxoplasma gondii* infection in immunocompromised patients: A systematic review and meta-analysis. Front Microbiol (2017) 8:389. doi: 10.3389/fmicb.2017.00389 28337191 PMC5343064

[4] Robert-Gangneux F Dardé ML. Epidemiology of and diagnostic strategies for toxoplasmosis. Clin Microbiol Rev (2012) 25(2):264–96. doi: 10.1128/CMR.05013-11 PMC3346298 22491772

[5] Bresciani KD da Costa AJ. Congenital Toxoplasmosis in Humans and Domestic Animals. Sharjah: Bentham Science Publishers (2018).

[6] Randall LM Hunter CA. Parasite dissemination and the pathogenesis of toxoplasmosis. Eur J Microbiol Immunol (Bp). (2011) 1(1):3–9. doi: 10.1556/EuJMI.1.2011.1.3 24466431 PMC3894809

[7] Yap GS Sher A. Cell-mediated immunity to *Toxoplasma gondii*: initiation, regulation and effector function. Immunobiology (1999) 201(2):240–7. doi: 10.1016/S0171-2985(99)80064-3 10631573

[8] Khan IA Moretto M. Immune responses to *Toxoplasma gondii* . Curr Opin Immunol (2022) 77:102226. doi: 10.1016/j.coi.2022.102226 35785567

[9] Bliss SK Marshall AJ Zhang Y Denkers EY. Human polymorphonuclear leukocytes produce IL-12, TNF-alpha, and the chemokines macrophage-inflammatory protein-1 alpha and -1 beta in response to *Toxoplasma gondii* antigens. J Immunol (1999) 162(12):7369–75. doi: 10.4049/jimmunol.162.12.7369 10358188

[10] Bliss SK Zhang Y Denkers EY. Murine neutrophil stimulation by *Toxoplasma gondii* antigen drives high level production of IFN-gamma-independent IL-12. J Immunol (1999) 163(4):2081–8. doi: 10.4049/jimmunol.163.4.2081 10438947

[11] Sturge CR Benson A Raetz M Wilhelm CL Mirpuri J Vitetta ES. TLR-independent neutrophil-derived IFN-γ is important for host resistance to intracellular pathogens. Proc Natl Acad Sci U S A. (2013) 110(26):10711–6. doi: 10.1073/pnas.1307868110 PMC3696766 23754402

[12] Biswas A French T Düsedau HP Mueller N Riek-Burchardt M Dudeck A. Behavior of neutrophil granulocytes during *Toxoplasma gondii* infection in the central nervous system. Front Cell Infect Microbiol (2017) 7:259. doi: 10.3389/fcimb.2017.00259 28680853 PMC5478696

[13] Bliss SK Butcher BA Denkers EY. Rapid recruitment of neutrophils containing prestored IL-12 during microbial infection. J Immunol (2000) 165(8):4515–21. doi: 10.4049/jimmunol.165.8.4515 11035091

[14] Coombes JL Charsar BA Han SJ Halkias J Chan SW Koshy AA. Motile invaded neutrophils in the small intestine of *Toxoplasma gondii*-infected mice reveal a potential mechanism for parasite spread. Proc Natl Acad Sci U S A. (2013) 110(21):E1913–22. doi: 10.1073/pnas.1220272110 PMC3666704 23650399

[15] Gregg B Taylor BC John B Tait-Wojno ED Girgis NM Miller N. Replication and distribution of *Toxoplasma gondii* in the small intestine after oral infection with tissue cysts. Infect Immun (2013) 81(5):1635–43. doi: 10.1128/IAI.01126-12 PMC3647985 23460516

[16] Brinkmann V Reichard U Goosmann C Fauler B Uhlemann Y Weiss DS. Neutrophil extracellular traps kill bacteria. Science (2004) 303(5663):1532–5. doi: 10.1126/science.1092385 15001782

[17] Fuchs TA Abed U Goosmann C Hurwitz R Schulze I Wahn V. Novel cell death program leads to neutrophil extracellular traps. J Cell Biol (2007) 176(2):231–41. doi: 10.1083/jcb.200606027 PMC2063942 17210947

[18] Pilsczek FH Salina D Poon KK Fahey C Yipp BG Sibley CD. A novel mechanism of rapid nuclear neutrophil extracellular trap formation in response to Staphylococcus aureus. J Immunol (2010) 185(12):7413–25. doi: 10.4049/jimmunol.1000675 21098229

[19] Yipp B Petri B Salina D Jenne CN Scott BNV Zbytnuik LD. Infection-induced NETosis is a dynamic process involving neutrophil multitasking in *vivo* . Nat Med (2012) 18:1386–93. doi: 10.1038/nm.2847 PMC4529131 22922410

[20] Saffarzadeh M Cabrera-Fuentes HA Veit F Jiang D Scharffetter-Kochanek K Gille CG. Characterization of rapid neutrophil extracellular trap formation and its cooperation with phagocytosis in human neutrophils. Discoveries (Craiova). (2014) 2(2):e19. doi: 10.15190/d.2014.11 32309548 PMC6941580

[21] Parker H Dragunow M Hampton MB Kettle AJ Winterbourn CC. Requirements for NADPH oxidase and myeloperoxidase in neutrophil extracellular trap formation differ depending on the stimulus. J Leukoc Biol (2012) 92(4):841–9. doi: 10.1189/jlb.1211601 22802447

[22] Rochael NC Guimarães-Costa AB Nascimento MT DeSouza-Vieira TS Oliveira MP Garcia e Souza LF. Classical ROS-dependent and early/rapid ROS-independent release of Neutrophil Extracellular Traps triggered by *Leishmania* parasites. Sci Rep (2015) 5:18302. doi: 10.1038/srep18302 26673780 PMC4682131

[23] Kenny EF Herzig A Krüger R Muth A Mondal S Thompson PR. Diverse stimuli engage different neutrophil extracellular trap pathways. Elife (2017) 6:e24437. doi: 10.7554/eLife.24437 28574339 PMC5496738

[24] Estúa-Acosta GA Zamora-Ortiz R Buentello-Volante B García-Mejía M Garfias Y. Neutrophil extracellular traps: current perspectives in the eye. Cells (2019) 8(9):979. doi: 10.3390/cells8090979 31461831 PMC6769795

[25] Abi Abdallah DS Lin C Ball CJ King MR Duhamel GE Denkers EY. *Toxoplasma gondii* triggers release of human and mouse neutrophil extracellular traps. Infect Immun (2012) 80(2):768–77. doi: 10.1128/IAI.05730-11 PMC3264325 22104111

[26] Reichel M Muñoz-Caro T Sanchez Contreras G Rubio García A Magdowski G Gärtner U. Harbour seal (Phoca vitulina) PMN and monocytes release extracellular traps to capture the apicomplexan parasite *Toxoplasma gondii* . Dev Comp Immunol (2015) 50(2):106–15. doi: 10.1016/j.dci.2015.02.002 25681075

[27] Yildiz K Gokpinar S Gazyagci AN Babur C Sursal N Azkur AK. Role of NETs in the difference in host susceptibility to *Toxoplasma gondii* between sheep and cattle. Vet Immunol Immunopathol (2017) 189:1–10. doi: 10.1016/j.vetimm.2017.05.005 28669381

[28] Yildiz K Gokpinar S Sursal N Babur C Ozen D Azkur AK. Extracellular trap formation by donkey polymorphonuclear neutrophils against *Toxoplasma gondii* . J Equine Vet Science. (2019) 73:1–9. doi: 10.1016/j.jevs.2018.11.002

[29] Lacerda LC Dos Santos JL Wardini AB da Silva AN Santos AG Silva Freire HP. *Toxoplasma gondii* induces extracellular traps release in cat neutrophils. Exp Parasitol (2019) 207:107770. doi: 10.1016/j.exppara.2019.107770 31586454

[30] Imlau M Conejeros I Muñoz-Caro T Zhou E Gärtner U Ternes K. Dolphin-derived NETosis results in rapid *Toxoplasma gondii* tachyzoite ensnarement and different phenotypes of NETs. Dev Comp Immunol (2020) 103:103527. doi: 10.1016/j.dci.2019.103527 31655127

[31] Karakurt G Yildiz K. *In vitro* investigation of NETosis reaction developing from dog polymorphonuclear neutrophils to *Toxoplasma gondii* . Turk J Vet Anim Sci (2020) 44(4):886–93. doi: 10.3906/vet-2002-6

[32] Macedo IS Lima MVA Souza JS Rochael NC Caldas PN Barbosa HS. Extracellular traps released by neutrophils from cats are detrimental to *Toxoplasma gondii* infectivity. Microorganisms (2020) 8(11):1628. doi: 10.3390/microorganisms8111628 33105542 PMC7716220

[33] Wei Z Wang Z Liu X Wang C Han Z Wu D. *Toxoplasma gondii* triggers neutrophil extracellular traps release in dogs. Front Cell Infect Microbiol (2020) 10:429. doi: 10.3389/fcimb.2020.00429 33102243 PMC7546211

[34] Simsek NS Cakmak A Yildiz K. *In vitro* investigation on extracellular traps formation of cat polymorphonuclear leucocytes against *Toxoplasma gondii* . Turk J Vet Anim. (2021) 45:873–80. doi: 10.3906/vet-2101-53

[35] Chen M Jin Z Jin Q Liu W Gao X Hong H. *Toxoplasma gondii* triggers heterophil extracellular traps *via* NADPH oxidase, ERK_1/2_ and P38 signalling pathways, glycolysis and autophagy in chickens. Parasite Immunol (2023) 45(8):e13001. doi: 10.1111/pim.13001 37340931

[36] Velásquez ZD Peixoto R Gärtner U Hermosilla C Taubert A Conejeros I. Dynamics of cell cycle proteins involved in *Toxoplasma gondii*-induced bovine NET formation. Front Immunol (2023) 14:1125667. doi: 10.3389/fimmu.2023.1125667 36875070 PMC9981159

[37] Miranda FJB Rocha BC Pereira MCA Pereira LMN de Souza EHM Marino AP. *Toxoplasma gondii*-induced neutrophil extracellular traps amplify the innate and adaptive response. mBio (2021) 12(5):e0130721. doi: 10.1128/mBio.01307-21 34607465 PMC8546543

[38] Hakkim A Fuchs TA Martinez NE Hess S Prinz H Zychlinsky A. Activation of the Raf-MEK-ERK pathway is required for neutrophil extracellular trap formation. Nat Chem Biol (2011) 7(2):75–7. doi: 10.1038/nchembio.496 21170021

[39] Schauer C Janko C Munoz LE Zhao Y Kienhöfer D Frey B. Aggregated neutrophil extracellular traps limit inflammation by degrading cytokines and chemokines. Nat Med (2014) 20(5):511–7. doi: 10.1038/nm.3547 24784231

[40] Lima TS Gov L Lodoen MB. Evasion of Human Neutrophil-Mediated Host Defense during *Toxoplasma gondii* Infection. mBio (2018) 9(1):e02027–17. doi: 10.1128/mBio.02027-17 PMC5821086 29440572

[41] Zhao Y Marple AH Ferguson DJ Bzik DJ Yap GS. Avirulent strains of *Toxoplasma gondii* infect macrophages by active invasion from the phagosome. Proc Natl Acad Sci U S A. (2014) 111(17):6437–42. doi: 10.1073/pnas.1316841111 PMC4035997 24733931

[42] Gonzalez AS Bardoel BW Harbort CJ Zychlinsky A. Induction and quantification of neutrophil extracellular traps. Methods Mol Biol (2014) 1124:307–18. doi: 10.1007/978-1-62703-845-4_20 24504961

[43] Lima TS Mallya S Jankeel A Messaoudi I Lodoen MB. *Toxoplasma gondii* extends the life span of infected human neutrophils by inducing cytosolic PCNA and blocking activation of apoptotic caspases. mBio (2021) 12(1):e02031–20. doi: 10.1128/mBio.02031-20 PMC7858050 33500339

[44] Papayannopoulos V Metzler KD Hakkim A Zychlinsky A. Neutrophil elastase and myeloperoxidase regulate the formation of neutrophil extracellular traps. J Cell Biol (2010) 191(3):677–91. doi: 10.1083/jcb.201006052 PMC3003309 20974816

[45] Leshner M Wang S Lewis C Zheng H Chen XA Santy L. PAD4 mediated histone hypercitrullination induces heterochromatin decondensation and chromatin unfolding to form neutrophil extracellular trap-like structures. Front Immunol (2012) 3:307. doi: 10.3389/fimmu.2012.00307 23060885 PMC3463874

[46] Favata MF Horiuchi KY Manos EJ Daulerio AJ Stradley DA Feeser WS. Identification of a novel inhibitor of mitogen-activated protein kinase kinase. J Biol Chem (1998) 273(29):18623–32. doi: 10.1074/jbc.273.29.18623 9660836

[47] Condliffe AM Davidson K Anderson KE Ellson CD Crabbe T Okkenhaug K. Sequential activation of class IB and class IA PI3K is important for the primed respiratory burst of human but not murine neutrophils. Blood (2005) 106(4):1432–40. doi: 10.1182/blood-2005-03-0944 15878979

[48] DeSouza-Vieira T Guimarães-Costa A Rochael NC Lira MN Nascimento MT Lima-Gomez PS. Neutrophil extracellular traps release induced by *Leishmania*: role of PI3Kγ, ERK, PI3Kσ, PKC, and [Ca2+]. J Leukoc Biol (2016) 100(4):801–10. doi: 10.1189/jlb.4A0615-261RR PMC5014744 27154356

[49] Wu YT Tan HL Shui G Bauvy C Huang Q Wenk MR. Dual role of 3-methyladenine in modulation of autophagy *via* different temporal patterns of inhibition on class I and III phosphoinositide 3-kinase. J Biol Chem (2010) 285(14):10850–61. doi: 10.1074/jbc.M109.080796 PMC2856291 20123989

[50] Pedra-Rezende Y Macedo IS Midlej V Mariante RM Menna-Barreto RFS. Different drugs, same end: ultrastructural hallmarks of autophagy in pathogenic protozoa. Front Microbiol (2022) 13:856686. doi: 10.3389/fmicb.2022.856686 35422792 PMC9002357

[51] Skendros P Mitroulis I Ritis K. Autophagy in neutrophils: from granulopoiesis to neutrophil extracellular traps. Front Cell Dev Biol (2018) 6:109. doi: 10.3389/fcell.2018.00109 30234114 PMC6131573

[52] Remijsen Q Vanden Berghe T Wirawan E Asselbergh B Parthoens E De Rycke R. Neutrophil extracellular trap cell death requires both autophagy and superoxide generation. Cell Res (2011) 21(2):290–304. doi: 10.1038/cr.2010.150 21060338 PMC3193439

[53] Park SY Shrestha S Youn YJ Kim JK Kim SY Kim HJ. Autophagy primes neutrophils for neutrophil extracellular trap formation during sepsis. Am J Respir Crit Care Med (2017) 196(5):577–89. doi: 10.1164/rccm.201603-0596OC 28358992

[54] Zhou E Conejeros I Velásquez ZD Muñoz-Caro T Gärtner U Hermosilla C. Simultaneous and positively correlated NET formation and autophagy in *Besnoitia besnoiti* tachyzoite-exposed bovine polymorphonuclear neutrophils. Front Immunol (2019) 10:1131. doi: 10.3389/fimmu.2019.01131 31191523 PMC6540735

[55] Brooks RL Bounous DI Andreasen CB. Functional comparison of avian heterophils with human and canine neutrophils. Comp Haematol Int (1996) 6:153–9. doi: 10.1007/BF00368459

[56] Nauseef WM. Human neutrophils ≠ murine neutrophils: Does it matter? Immunol Rev (2023) 314(1):442–56. doi: 10.1111/imr.13154 PMC10049967 36380497

[57] Orellana MA Suzuki Y Araujo F Remington JS. Role of beta interferon in resistance to *Toxoplasma gondii* infection. Infect Immun (1991) 59(9):3287–90. doi: 10.1128/iai.59.9.3287-3290.1991 PMC258166 1908831

[58] Gazzinelli RT Wysocka M Hieny S Scharton-Kersten T Cheever A Kühn R. In the absence of endogenous IL-10, mice acutely infected with *Toxoplasma gondii* succumb to a lethal immune response dependent on CD4+ T cells and accompanied by overproduction of IL-12, IFN-gamma and TNF-alpha. J Immunol (1996) 157(2):798–805. doi: 10.4049/jimmunol.157.2.798 8752931

[59] Suzuki Y Sher A Yap G Park D Neyer LE Liesenfeld O. IL-10 is required for prevention of necrosis in the small intestine and mortality in both genetically resistant BALB/c and susceptible C57BL/6 mice following peroral infection with *Toxoplasma gondii* . J Immunol (2000) 164(10):5375–82. doi: 10.4049/jimmunol.164.10.5375 10799901

[60] Kelly MN Kolls JK Happel K Schwartzman JD Schwarzenberger P Combe C. Interleukin-17/interleukin-17 receptor-mediated signaling is important for generation of an optimal polymorphonuclear response against *Toxoplasma gondii* infection. Infect Immun (2005) 73(1):617–21. doi: 10.1128/IAI.73.1.617-621.2005 PMC538931 15618203

[61] Mahmoud ME Ui F Salman D Nishimura M Nishikawa Y. Mechanisms of interferon-beta-induced inhibition of *Toxoplasma gondii* growth in murine macrophages and embryonic fibroblasts: role of immunity-related GTPase M1. Cell Microbiol (2015) 17(7):1069–83. doi: 10.1111/cmi.12423 25628099

[62] Moroda M Takamoto M Iwakura Y Nakayama J Aosai F. Interleukin-17A-Deficient mice are highly susceptible to *Toxoplasma gondii* infection due to excessively induced *T. gondii* HSP70 and interferon gamma production. Infect Immun (2017) 85(12):e00399–17. doi: 10.1128/IAI.00399-17 PMC5695131 28893913

[63] Hoppenbrouwers T Autar ASA Sultan AR Abraham TE van Cappellen WA Houtsmuller AB. *In vitro* induction of NETosis: Comprehensive live imaging comparison and systematic review. PloS One (2017) 12(5):e0176472. doi: 10.1371/journal.pone.0176472 28486563 PMC5423591

[64] Naccache PH Fernandes MJ. Challenges in the characterization of neutrophil extracellular traps: The truth is in the details. Eur J Immunol (2016) 46(1):52–5. doi: 10.1002/eji.201546022 26635275

[65] Petretto A Bruschi M Pratesi F Croia C Candiano G Ghiggeri G. Neutrophil extracellular traps (NET) induced by different stimuli: A comparative proteomic analysis. PloS One (2019) 14(7):e0218946. doi: 10.1371/journal.pone.0218946 31283757 PMC6613696

[66] Chapman EA Lyon M Simpson D Mason D Beynon RJ Moots RJ. Caught in a trap? Proteomic analysis of neutrophil extracellular traps in rheumatoid arthritis and systemic lupus erythematosus. Front Immunol (2019) 10:423. doi: 10.3389/fimmu.2019.00423 30915077 PMC6421309

[67] Ríos-López AL González GM Hernández-Bello R Sánchez-González A. Avoiding the trap: Mechanisms developed by pathogens to escape neutrophil extracellular traps. Microbiol Res (2021) 243:126644. doi: 10.1016/j.micres.2020.126644 33199088

[68] Guimarães-Costa AB DeSouza-Vieira TS Paletta-Silva R Freitas-Mesquita AL Meyer-Fernandes JR Saraiva EM. 3'-nucleotidase/nuclease activity allows *Leishmania* parasites to escape killing by neutrophil extracellular traps. Infect Immun (2014) 82(4):1732–40. doi: 10.1128/IAI.01232-13 PMC3993383 24516114

