# Supplementary material for: Human neutrophil extracellular traps do not impair in vitro Toxoplasma gondii infection
Source: Front Immunol. 2023 Dec 5;14:1282278. doi: 10.3389/fimmu.2023.1282278 (PMC10728484; doi:10.3389/fimmu.2023.1282278)
Supplement: Supplementary file 1 [file DataSheet_1.pdf]

Supplementary Figures

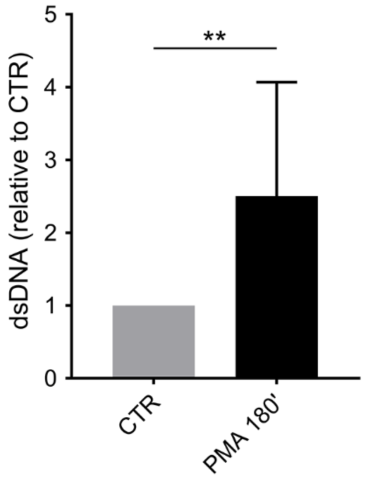

SUPPLEMENTARY FIGURE 1 | Human neutrophils release NETs in response to PMA. Neutrophils from healthy donors were incubated for 180 min with PMA (100 nM). Supernatants were collected and released dsDNA was quantified with PicoGreen Kit ($n = 13$). Results are shown as mean (SD) relative to non-stimulated neutrophils (CTR). ** $p < 0.01$.

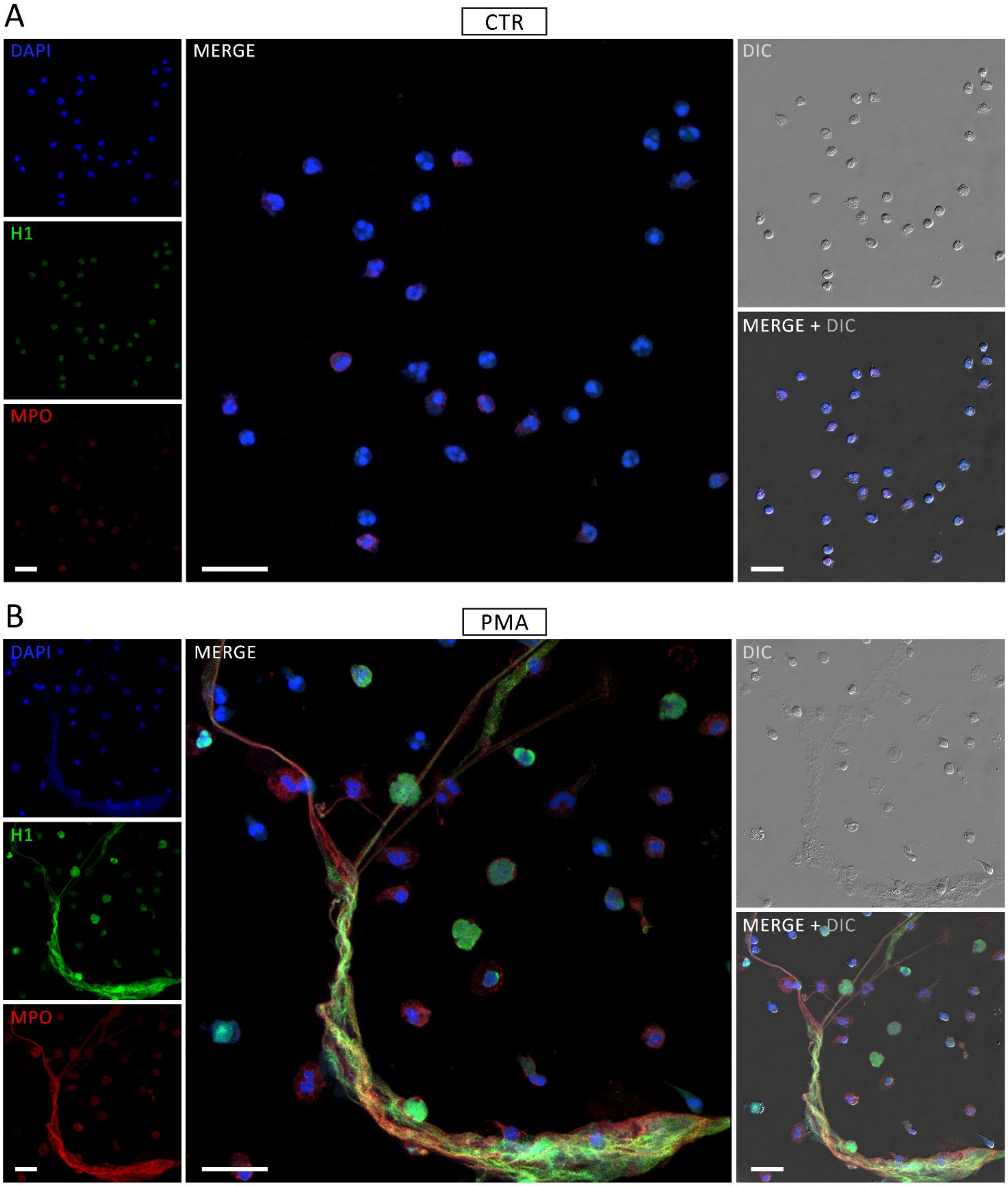

SUPPLEMENTARY FIGURE 2 | Classical NETs induced by PMA. Human neutrophils were incubated (PMA) or not (CTR) for 180 min PMA (100 nM), fixed and stained for histone H1 (*green*) and myeloperoxidase (MPO, *red*). DNA was counterstained with DAPI (*blue*). Bars = 30 µm.

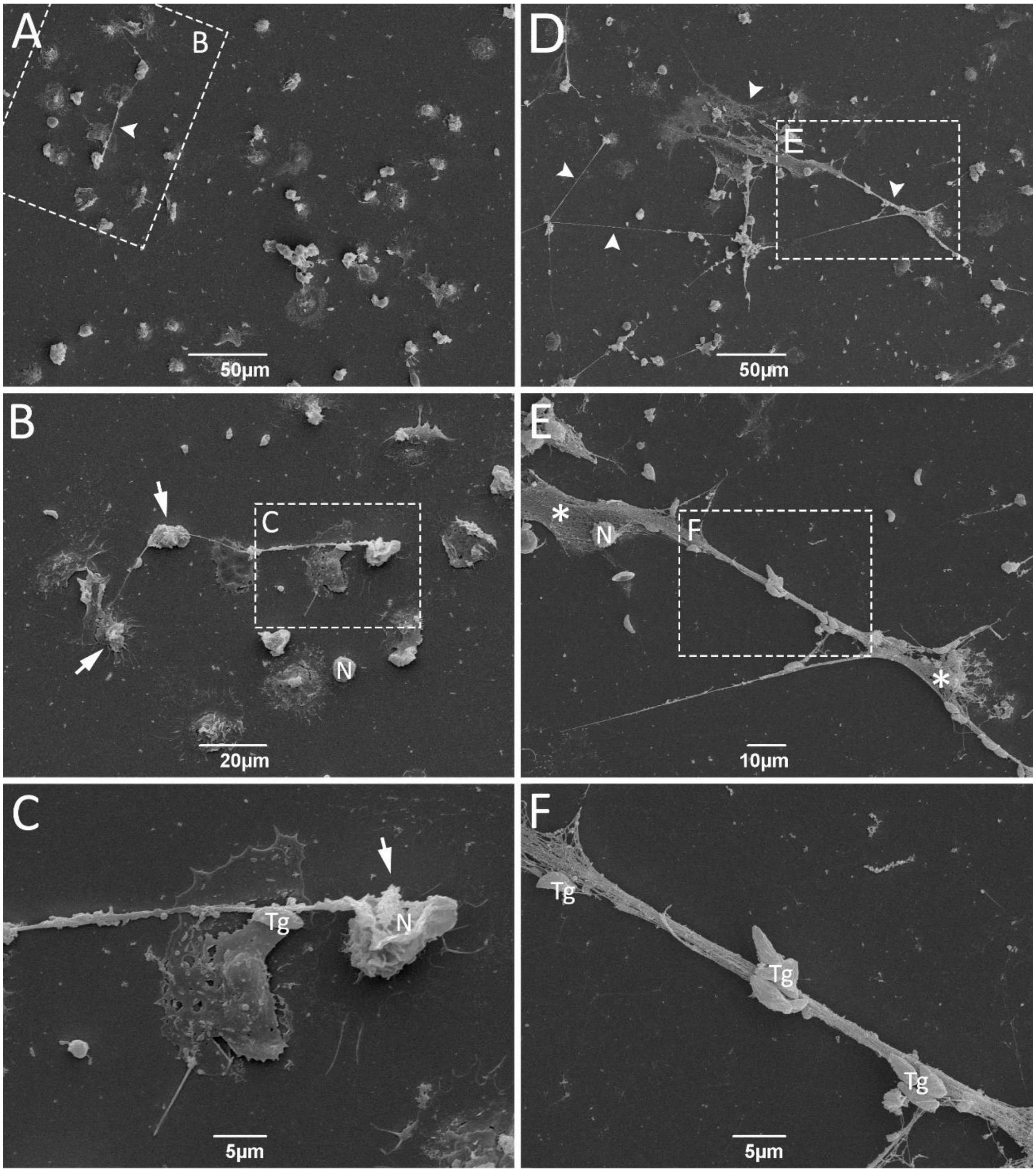

SUPPLEMENTARY FIGURE 3 | Ultrastructure of rapid and classical NETs induced by *T. gondii* tachyzoites as seen by SEM. Human neutrophils were incubated for 15 min (**A**–**C**) or 180 min (**D**–**F**) with RH strain tachyzoites (5:1 parasites:neutrophil ratio) and processed for SEM. NETs are indicated by arrowheads (**A**, **D**). Neutrophils with healthy appearance releasing NETs are indicated by arrows (**B**, **C**), while structures resembling dead neutrophils that originated NETs are designated by asterisks (E). Tg = *T. gondii*; N = neutrophil.

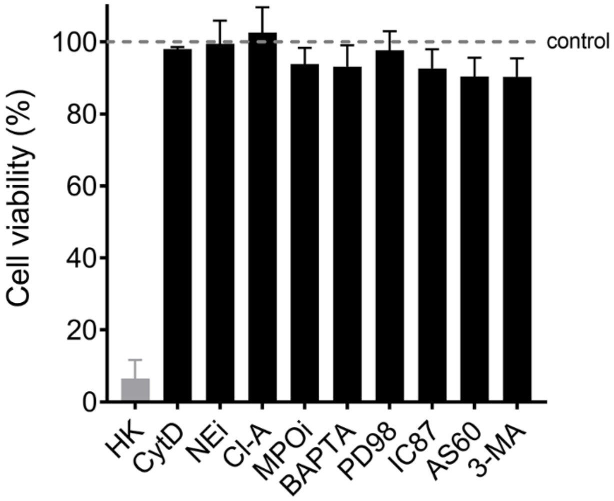

SUPPLEMENTARY FIGURE 4 | Viability of neutrophils after treatment with inhibitors. The cytotoxicity of the inhibitors to neutrophils was examined with PrestoBlue Cell Viability Reagent. After treatment with compounds, PrestoBlue was added 20 min before the end of the incubation time. Analysis was performed on a microplate reader using excitation/emission wavelengths of 560/590 nm (n = 2–5). Data are presented as percentage of control and are shown as means (SEM). HK = heat killed neutrophils.

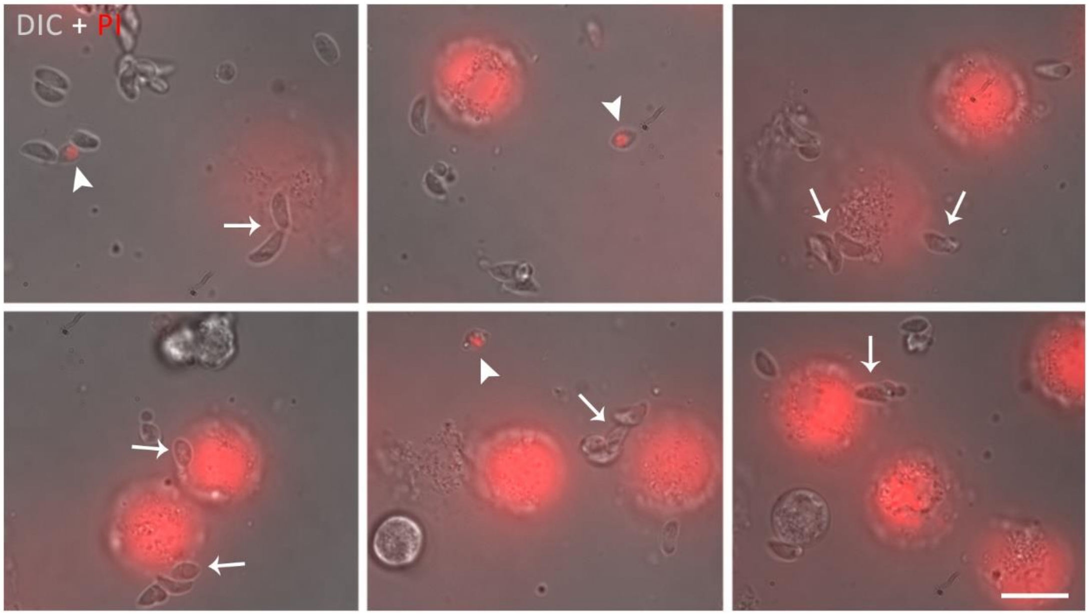

SUPPLEMENTARY FIGURE 5 | Viability assessment of *T. gondii* in contact with NETs. Human neutrophils were stimulated with *T. gondii* tachyzoites of the RH strain (5:1 parasites:neutrophil ratio) and incubated with PI (3 µg/ml). At the end of the experiment (270 min), random fields were photographed, indicating parasites stained with PI without contact with NET (white arrowhead) and live parasites in contact with NET (white arrow). Bar = 10 µm.

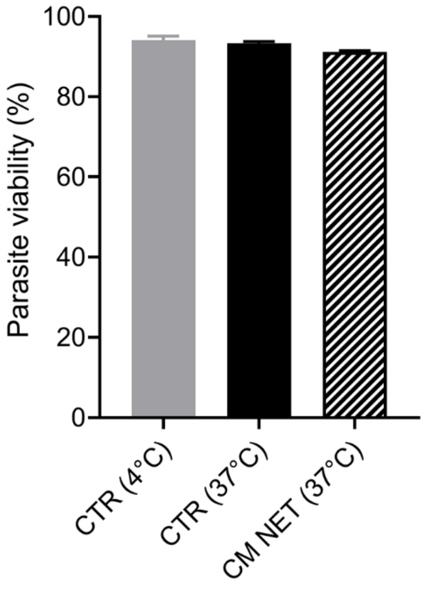

SUPPLEMENTARY FIGURE 6 | Viability assessment of *T. gondii* tachyzoites. The parasites were kept either at 4°C or under conditions similar to the videomicroscopy assay (37°C) for a duration of 270 min, and their viability was determined through PI staining. $n = 2$.
